# Induction of Mouse Melioidosis with Meningitis by CD11b^+^ Phagocytic Cells Harboring Intracellular *B. pseudomallei* as a Trojan Horse

**DOI:** 10.1371/journal.pntd.0002363

**Published:** 2013-08-08

**Authors:** Pei-Ju Liu, Yao-Shen Chen, Hsi-Hsu Lin, Wei-Feng Ni, Tsung-Han Hsieh, Hsu-Tzu Chen, Ya-Lei Chen

**Affiliations:** 1 Department of Biotechnology, National Kaohsiung Normal University, Kaohsiung, Taiwan; 2 Division of Infectious Diseases, Kaohsiung Veterans General Hospital, Kaohsiung, Taiwan; 3 Department of Internal Medicine, National Yang-Ming University, Taipei, Taiwan; 4 Graduate Institute of Environmental Education, National Kaohsiung Normal University, Kaohsiung, Taiwan; 5 Department of Infectious Disease, E-DA Hospital/I-Shou University, Kaohsiung, Taiwan; Mahidol University, Thailand

## Abstract

**Background:**

Approximately 3–5% of patients with melioidosis manifest CNS symptoms; however, the clinical data regarding neurological melioidosis are limited.

**Methods and Findings:**

We established a mouse model of melioidosis with meningitis characterized by neutrophil infiltration into the meninges histologically and *B. pseudomallei* in the cerebrospinal fluid (CSF) by bacteriological culturing methods. As the disease progresses, the bacteria successively colonize the spleen, liver, bone marrow (BM) and brain and invade splenic and BM cells by days 2 and 6 post-infection, respectively. The predominant cell types intracellularly infected with *B. pseudomallei* were splenic and BM CD11b^+^ populations. The CD11b^+^Ly6C^high^ inflamed monocytes, CD11b^+^Ly6C^low^ resident monocytes, CD11b^+^Ly6G^+^ neutrophils, CD11b^+^F4/80^+^ macrophages and CD11b^+^CD19^+^ B cells were expanded in the spleen and BM during the progression of melioidosis. After adoptive transfer of CD11b populations harboring *B. pseudomallei*, the infected CD11b^+^ cells induced bacterial colonization in the brain, whereas CD11b^−^ cells only partially induced colonization; extracellular (free) *B. pseudomallei* were unable to colonize the brain. CD62L (selectin) was absent on splenic CD11b^+^ cells on day 4 but was expressed on day 10 post-infection. Adoptive transfer of CD11b^+^ cells expressing CD62L (harvested on day 10 post-infection) resulted in meningitis in the recipients, but transfer of CD11b^+^ CD62L-negative cells did not.

**Conclusions/Significance:**

We suggest that *B. pseudomallei*-infected CD11b^+^ selectin-expressing cells act as a Trojan horse and are able to transmigrate across endothelial cells, resulting in melioidosis with meningitis.

## Author Summary

A mouse model of melioidosis with meningitis was established in this study by injecting mice with *B. pseudomallei* intravenously. Bacteria successively colonized the spleen, liver, bone marrow (BM) and brain. After infection, the splenic and BM CD11b^+^ populations carrying intracellular *B. pseudomallei* selectively expanded and became predominant. After adoptive transfer, melioidosis with meningitis was induced by the infected BM CD11b^+^ cells, partially induced by BM CD11b^−^ cells and was not induced by splenic CD11b^−^ cells or extracellular bacteria. The induction of melioidosis with meningitis was correlated with an increase in splenic CD11b^+^ selectin (CD62L)-expressing cells.

## Introduction

The saprophytic rod *Burkholderia pseudomallei* is a causative agent of melioidosis and is endemic to tropical areas such as Southeast Asia and northern Australia [1]. The main modes of transmission of melioidosis are inhalation and subcutaneous inoculation [2]. Ingestion can cause a systemic infection, and consequently, the gastrointestinal tract can serve as a reservoir for the dissemination of melioidosis [3], [4]. Acute melioidosis with septicemia, which is transmitted through various routes of infection, is the most severe for humans [5] and animals [3], [6]–[10]. However, the clinical spectrum of melioidosis varies; approximately 3–5% of patients develop neurological symptoms, including macroscopic brain abscess, brainstem encephalitis or flaccid paraparesis [11]–[15]. Although melioidosis with primary meningitis is rarely seen, meningitis could arise due to the spread of *B. pseudomallei* from a remote infected site *via* the blood-stream or from ruptured cerebral abscesses into adjacent foci [15]. Fatalities due to melioidosis with meningitis have been reported in neonates, patients receiving inappropriate antibiotic treatment and patients with long-term infections [14], [16]–[18].

During mouse bacteremic melioidosis, the spleen and liver are the primary infected foci; both contain a large amount of *B. pseudomallei*, even during chronic melioidosis [7], [10], [19]. It has been reported that the bacteria colonizing the lungs, lymph nodes and brain disseminate locally from infected foci [7], [20]. The induction of interferon-γ (IFN-γ) and the activation of phagocytic cells are essential for the early control of *B. pseudomallei* in mice. IFN-γ depletion in the blood results in a rapid increase in bacterial burdens in the organs [7], [21]. The replication of invasive *B. pseudomallei* in infected foci can be controlled by host immunological events that recruit a large number of activated neutrophils and monocytes [19], [22], [23]. However, it is very difficult for the host to clear *B. pseudomallei* because *B. pseudomallei* invades macrophages, monocytes and hepatocytes and grows intracellularly [24]–[26]. *B. pseudomallei* induces cellular actin polymerization and rearrangement, resulting in cell-cell fusion and the formation of multinucleate giant cells, thus facilitating cell-to-cell spread [27]–[29]. It is believed that the intracellular bacteria grow steadily when host cytokines are depleted or when macrophage activity is attenuated [30], [31].

Meningeal neutrophil infiltration is a hallmark of bacterial meningitis. Leukocytes do not normally adhere to endothelial cells except during activation. Endothelial cells and leukocytes express complementary adhesion molecules (selectins and integrins) that are responsible for rolling, adhesion and transendothelial migration (of leukocytes) into the meninges [32]–[34]. Mouse bacteremic melioidosis induces a robust inflammatory response marked by the upregulation of the cytokine-induced neutrophil chemoattractant (KC), macrophage inflammatory protein-2 (MIP-2), monocyte chemoattractant protein-1 (MCP-1), granulocyte-macrophage colony-stimulating factor (GM-CSF) and macrophage CSF (M-CSF) [19]. Circulating activated phagocytes that are intracellularly infected with *B. pseudomallei* can cross the endothelial cells into the brain, and consequently, melioidosis-associated meningitis can occur. In this study, we addressed whether an activated phagocytic population harboring *B. pseudomallei* plays a role in inducing mouse melioidosis with meningitis.

## Methods

### Ethics statement

In this study, animal experiments were conducted following the Guide for the Care and Use of Laboratory Animals (National Animal Laboratory Center, Taiwan) and were approved by the Institutional Animal Care and Use Committee at the National Kaohsiung Normal University, Taiwan (approval ID: 9901). Linking *B. pseudomallei* data and private information of melioidosis patients is legally prohibited by the Personal Information Protection Act (Taiwan). All experiments using viable *B. pseudomallei* were performed in an air flow-controlled lab (BSL III level), and the procedures were approved by the Institutional Biosafety Committee (NKNU, Taiwan).

### Strains and plasmids

*B. pseudomallei* vgh19 (id, 3052; http://bpseudomallei.mlst.net) was obtained from the blood of a melioidosis patient with septicemia in Kaohsiung Veterans General Hospital, Taiwan. *B. pseudomallei-*GFP (expressing green fluorescence protein [GFP]) was made using a tri-parental mating system. The plasmid pKNOCK (*wbiI*-p*fliC*-*gfp*-*cat*) was constructed using specific PCR products: the truncated *wbiI* gene (ID: 3689613) and the *fliC* promoter [p*fliC*, 517 bp upstream of the coding region of *fliC* gene (ID:3688602)] from *B. pseudomallei* vgh19. The *gfp* gene was excised from the pUT-miniTn5-*gfp*-*tet* (AY364166) plasmid using appropriate restriction sites [35]. For conjugation, *E. coli* DH5α *pir* pKNOCK (*wbiI*-p*fliC*-*gfp*-*cat*, Cm^r^,Ap^s^; 10^9^ CFU) was used as a donor strain, *E. coli* pRK2013 (10^9^ CFU; Cm^s^, Ap^s^) was used as a helper bacteria and *B. pseudomallei* vgh19 was the recipient (10^9^ CFU; Cm^s^, Ap^r^); the three strains were mixed and filtered onto cellulose paper (<0.45 µm). *B. pseudomallei*-GFP was selected for using the chloramphenicol resistance and screened for using green fluorescence observed by fluorescent microscopy (Eclipse 50i; Nikon, Shinjuku, Tokyo, Japan). The specific insertion site was confirmed by PCR, sequencing and Southern hybridization. The genome of the GFP strain contained a single recombination event and generated an insertion of the p*fliC*-*gfp*-*cat* cassette into the *wbiI* gene, creating a defective *wbiI* gene and a functional *wbiI* gene (Figure S1).

### Induction of mouse melioidosis

BALB/c mice (females, 8 weeks old; n = 20 for each group) were intravenously infected *via* the tail vein with *B. pseudomallei* vgh19 or -GFP at the indicated concentration (100 µl; 5–5×10^5^ CFU). Survival rates and disease scores were recorded daily. The clinical scores including neurological signs are described in Table S1.

### Cytokines and liver function enzymes

At days 0, 2, 5 and 10 post-infection, serum was obtained from blood collected from each mouse by cardiac puncture to analyze cytokines and liver enzyme function (n = 6 for each experimental group, performed in duplicate). The liver enzymes glutamic oxaloacetic transaminase (GOT) and glutamic pyruvic transaminase (GPT) were measured using a clinical biochemistry analysis system (COBAS INTEGRATM 800, Roche, Basel, Switzerland).

The mouse serum cytokines TNF-α, IL-6, IL-12, IFN-γ, IL-10 and MCP-1 were measured using a Cytometric Bead Array kit (CBA; BD Biosciences, San Diego, CA, USA) according to the manufacturer's instructions [36]. Two-color cytometric data were collected at emission wavelengths of 423 nm and 578 nm by flow cytometry (BD Biosystems FACSCalibur system, BD Biosciences). Data analysis was performed using FCAP Array Software (version, 3.0; Bender Medsystems, Burlington, CA, USA), and standard curves were generated for each cytokine using the mixed cytokine/chemokine standard provided.

### Histological examination

The spleen, liver, bone marrow (BM) and skull were excised, fixed in 4% formaldehyde, de-calcified with 10% trichloroacetic acid if the tissue contained bone and processed for paraffin wax embedding using standard techniques [37].

### Bacterial burdens

At the indicated times, the infected mice were sacrificed, and the solid organs (spleen, liver and brain) were excised. Bacterial loads (CFU/g) in the liver (*ca.* 0.5 g), spleen (*ca.* 0.02 g) and brain (*ca.* 0.4 g) were determined using a sequential weighing, homogenization in 500 µl PBS (phosphate buffered saline) and serial dilution protocol [38]. To remove contaminating intravascular blood and bacteria, the mice were perfused with PBS by cardiac injection (*ca.* 100 ml), and the bacterial burdens in the brain were determined. Blood (100 µl) was collected from the heart by cardiac puncture, and the bacterial concentration (CFU/ml) was measured by the serial dilution method followed by plate counts. To cultivate *B. pseudomallei*, both blood (100 µl) and CSF (cerebrospinal fluid, 5 µl) were seeded into culture bottles containing Ashdown's broth. CSF was collected from the cisterna magna by capillary (0.05 mm) puncture; the samples were discarded if contaminated with red blood cells. After a 7 d-incubation, the presence or absence of *B. pseudomallei* was observed. BM was aseptically isolated from the femur by removing both ends of the bone and flushing the interior 1 ml PBS/2% FCS (fetal calf serum) using a syringe. After flushing, the total numbers of bacteria (CFU/ml) in the BM were determined using serial dilution and plate counts. Bacterial burden data were averaged (mean ± standard deviation (SD)) from independent experiments (n = 6) within each condition in duplicate. If the bacterial load in the brain was very low as anticipation, the entire brain homogenate was plated. The limits of detection were 20, 50 and 3 CFU/g for liver, spleen and brain and 10 CFU/ml for blood and BM. The lower limit of CSF detection was 200 CFU/ml in this study.

### Preparation of single cell suspensions and determination of intracellular bacterial amounts

Single cell suspensions from spleen and BM were prepared from infected mice at the indicated times (n = 3 for each experimental group performed in duplicate). Mononuclear cell layers in the spleen were separated by Ficoll-hypaque density gradient centrifugation (Sigma, St Louis, MO, USA). Red blood cells were lysed by treating with 0.83% NH_4_Cl for 3 min and then rapidly buffering with PBS containing 2% FCS. BM suspensions were prepared from the liquid obtained from flushing after filtering using nylon membranes (30 µm; Millipore, Billerica, MA, USA). All preparations were treated with kanamycin (400 µg/ml for 2 h) to remove extracellular *B. pseudomallei*. The number of splenic and BM cells harboring *B. pseudomallei* (CFU/10^6^ cells, individual groups, n = 3, duplicate) was measured by plate counts after serial dilution.

### Determination of the subpopulations of spleen and BM

The single cell spleen and BM preparations were obtained as described above. Peripheral blood mononuclear cells (PBMCs) were prepared using OptiLyse C solution (Beckman Coulter, Inc., Brea, CA, USA). These cells were stained with monoclonal PE (phycoerythrin)- or PE-Cy 7-conjugated anti-mouse specific antibodies (Table S2 contains the clones, isotype controls, species and concentration of antibody used in this study) for 30 min on ice in the dark according to the manufacturer's protocol. Stained cells were evaluated by flow cytometry (Cell Lab Quanta SC, Beckman Coulter, Inc.), and the flow cytometry data were analyzed using CXP software (Beckman Coulter, Inc.).

### Isolation of subpopulations from spleen and BM

The CD11b^+^, Ly6C^+^ and CD19^+^ populations were isolated from the spleen or BM of infected mice using an EasySep Mouse Positive Selection Kit (n = 3 for each experimental group, duplicate; STEMCELL Tech. Inc., Vancouver, Canada). For each purification, the purity was confirmed to be >98% by flow cytometry. The CD11b^−^ population was collected from the unbound fraction from cells repeatedly passed through a CD11b^+^ cell binding column and was >83% pure. The amounts of intracellular *B. pseudomallei* (CFU) in each population, if necessary, were determined by the serial dilution method as described above.

### Adoptive transfer

For adoptive transfer, the donor cells in this study were splenic or BM, CD11b^+^ or CD11b^−^ and selectin-negative or selectin–expressing cells. CD11b^+^ or CD11b^−^ splenic or BM cells were isolated from the mice on day 10 post-infection. The selectin-negative cells were prepared from mouse spleens on day 4 post-infection while selectin-expressing cells were prepared on day 10 post-infection. The isolation protocols for single cell suspensions or CD11b subpopulations from spleen and BM were performed as described above. Prior to adoptive transfer, the donor cells (*ca.* 10^4^–10^6^ cells) were adjusted to contain 50, 500 or 2000 CFU of cultivated *B. pseudomallei*. This adjustment based on the ratio of cultivatable bacteria to fluorescent cells (refer to Text S1, protocols and comments of adjusting donor cells and Figure S2, Scheme of the adoptive transfer protocols). The data were unavailable if intracellular *B. pseudomallei* within the donor cells used for adoptive transfer were determined to be >±15% of 50, 500 or 2000 CFU by plate counts. These donor cells were adoptively transferred to recipient mice (uninfected BALB/c mice, female, 8–10 week old; n = 3 or 6 for each experimental condition, duplicate) by intravenous injection. On day 2 post-transfer, the mice were sacrificed and the bacterial burdens in the brain, liver, spleen and BM, the bacterial cultures using blood and CSF, the concentration of serum cytokine (TNF-α, IL-1β, IL-6, IFN-γ, MCP-1) and the levels of liver enzymes (GOT and GPT) in sera were determined by the protocols described above.

### Evaluation of mouse melioidosis with meningitis after adoptive transfer

Melioidosis with meningitis after adoptive transfer was evaluated by histological and bacteriological examination. On day 2 post-transfer, a total of 8 wax blocks containing brain tissue pieces cut from certain position for each skull were prepared from the mouse groups, including adoptive transfer of infected CD11b^+^, CD11b^−^, selectin-expressing and selectin-negative cells, respectively (n = 3 for each group, duplicate). After H&E staining (see above), histological changes were divided into intensive neutrophil infiltration (cellular debris), moderate neutrophil infiltration (no cellular debris) and vascular congestion with or without slight cell infiltration. The histological category of the recipient was determined according to their sections present in the most severe pathological features. Bacteriological examination was performed by collecting mouse CSF (5 µl) and testing for infection using bacterial culture techniques (as described above). After a 7-d incubation, the proportion (%) of samples with *B. pseudomallei* growth were calculated based on the outcomes of all tested mice.

### Statistical analysis

All data are expressed as the means ± SD and were derived from independent mice (n = 3 or n = 6), performed in two independent experiments. Differences between two experimental groups were analyzed using an unpaired Student's *t* test (SPSS, version 19.0); *p-values*<0.05 were considered to be statistically significant.

### Accession numbers

The nucleotide sequences obtained in this study are available at the National Center for Biotechnology Information (NCBI) under the following accession numbers: *wbiI* (gene ID: 3689613), *fliC* promoter (517 bp upstream of the coding region of *fliC*, gene ID:3688602) and pUT-miniTn5-*gfp*-*tet* (GenBank: AY364166). The nucleotide sequence of the plasmid pKNOCK-Cm can be found in reference 35 or at http://gordonlab.wustl.edu/plasmids/pKNOCK-cm%20sequence.txt.

## Results

### Mouse melioidosis with neurological symptoms

After intravenous injection of *B. pseudomallei* vgh19 (50 CFU), the survival of mice with melioidosis has two periods where death occurred (decline phases). Approximately 20% of the infected mice died within 4 days; the survivors rapidly declined and died on day 10 post-infection, with the exception of one mouse that died between 5 and 9 days (Figure S3). In this study, the overall progress of melioidosis was divided into three phases and their characteristics are summarized in Table S3.

During phase I, mice manifesting melioidosis developed a slightly hunched back, ruffled fur and showed decreased activity. The average clinical score was 6.56 (95% confidence interval [CI], 5.11–9.6). During phase II, the score decreased slightly to 4.2 (95% CI, 3.32–6.1); however, during phase III, 86.6% (13/15) of the mice developed severe disease with neurological signs (photophobia, ataxia and limb paresis or paralysis) as well as a rapid loss of body weight (up to 20% of the baseline) and a severely diminished activity level (clinical score, 18.5; 95% CI, 17.76–20.47). Cytokine levels (TNF-α, IL-6, IFN-γ, IL-10 and MCP-1, except for IL-12; Figure S4A) and liver enzyme activities (GOP and GPT; Figure S4B) in the infected mice were relatively high during phase I but lower during phase II. The levels of IFN-γ and MCP-1, both of which are involved in the activation and attraction of macrophages, increased during phase III.

**Figure 1:**
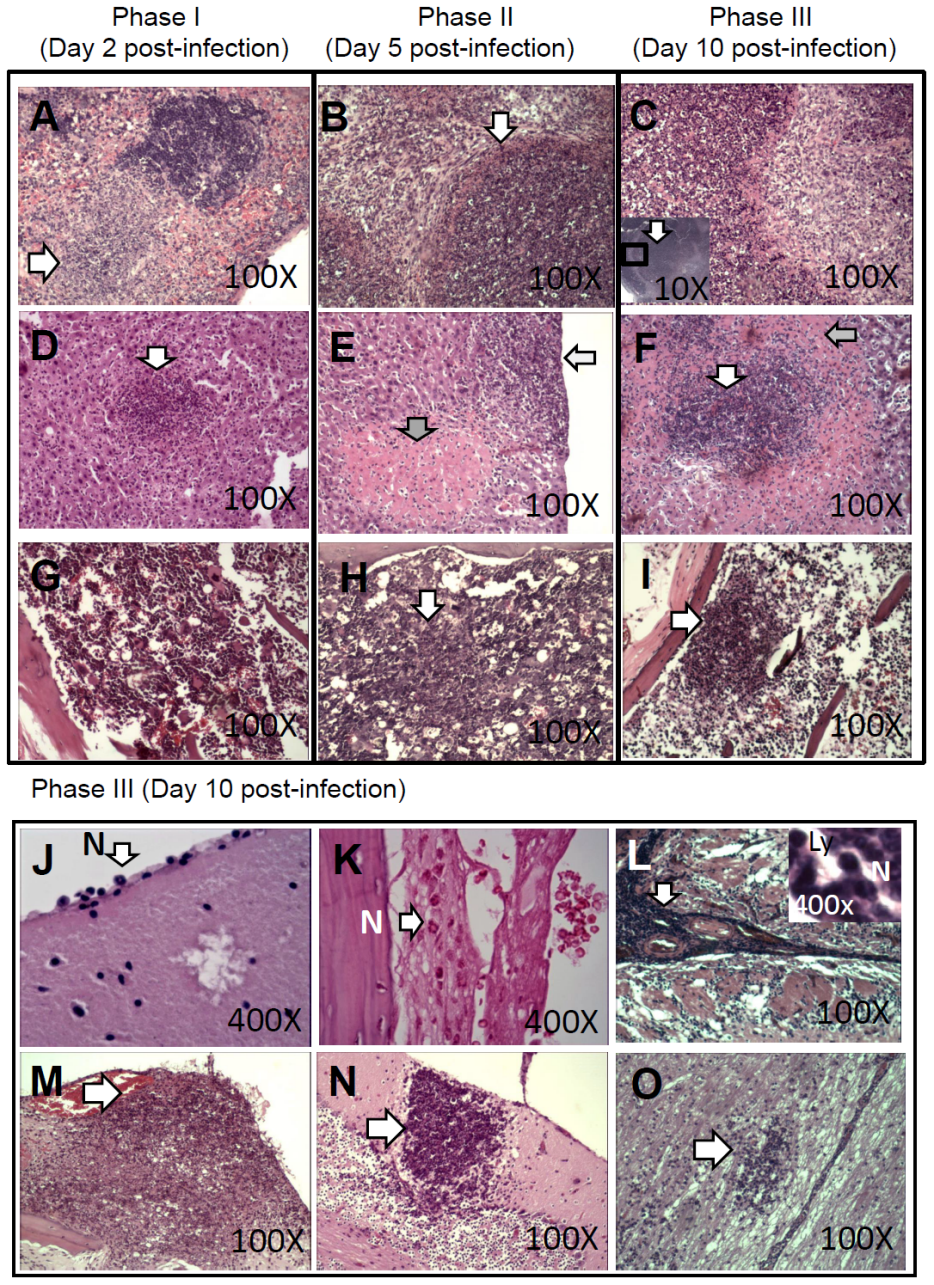
Histological examination. H&E staining of infected tissue was performed on days 2 (phase I), 5 (phase II) and 10 (phase III). Based on observations of the spleen, abscesses (indicated by arrows) occurred during phases I (A, 100×), II (B, 100×) and III (C, 100×). The lesions became very large during phase III (magnification, 10×; lower left corner of C). Based on observations of the liver, abscesses consisting of degraded neutrophils and cellular debris (indicated by white arrows) occurred during phases I (D, 100×), II (E, 100×) and III (F, 100×). Necrotic hepatocytes (indicated by gray arrows) were present in phase II and expanded during phase III. Normal hematogenous cells were observed in the BM during phase I (G, a representative femur image; 100×), and cellular debris (indicated by arrows) was present in the femur and vertebrae during phases II (H, a representative vertebral image; 100×) and III (I, a representative femur image; 100×). Because brain histological changes did not occur during phases I and II, only representative images of sections prepared during phase III are shown, including neutrophil (N) migration in cerebral margins (J, 400×), neutrophil infiltration in the meningeal subarachnoid space (K, 400×) and the accumulation of darkly stained cells (indicated by arrows) in the meninges (L, 100×). Using high magnification, both neutrophils (N) and lymphocytes (Ly) were observed (400×; upper right corner in L). Severe histopathological changes (indicated by arrows), including suppurative meningitis (M, 100×), cerebellar microabscess (N, 100×) and brain stem encephalitis (O, 100×), are indicated.

Histological examination of infected mice sacrificed on days 2 (phase I), 5 (phase II) and 10 (phase III) post-infection were performed. Multiple microabscesses consisting primarily of degraded neutrophils and cellular debris were observed in the spleen (Figure 1A–C) and liver (Figure 1D–F) during all phases. During the progression of melioidosis, abscesses in both of these organs increased and expanded. Necrotic hepatocytes were present in phase II and expanded during phase III. Although normal hematogenous cells were found in the BM during phase I, cellular debris was present in the femur and the vertebrae during both phase II and III (Figure 1G–I). Immunological activity occurred in the central nervous system (CNS) during phase III as determined by histology. In the mice evaluated during phase III (n = 15), minimal neutrophil migration into the cerebral margins was noted in 86.7% (n = 13) of the infected mice (Figure 1J), and neutrophil infiltration into the meningeal subarachnoid space was observed in 86.7% (n = 13) of the mice (Figure 1K). Severe meningitis was found in 73.3% (n = 11) of mice, as indicated by darkly stained cells (neutrophils, lymphocyte, microglia or unidentified cells) accumulating in the meninges (Figure 1L). Suppurative meningitis (n = 5; Figure 1M), cerebellar microabscesses (n = 2; Figure 1N) and brain stem encephalitis (n = 3; Figure 1O) were occasionally observed.

### Bacteria successively colonized the spleen, liver, BM and brain

**Figure 2:**
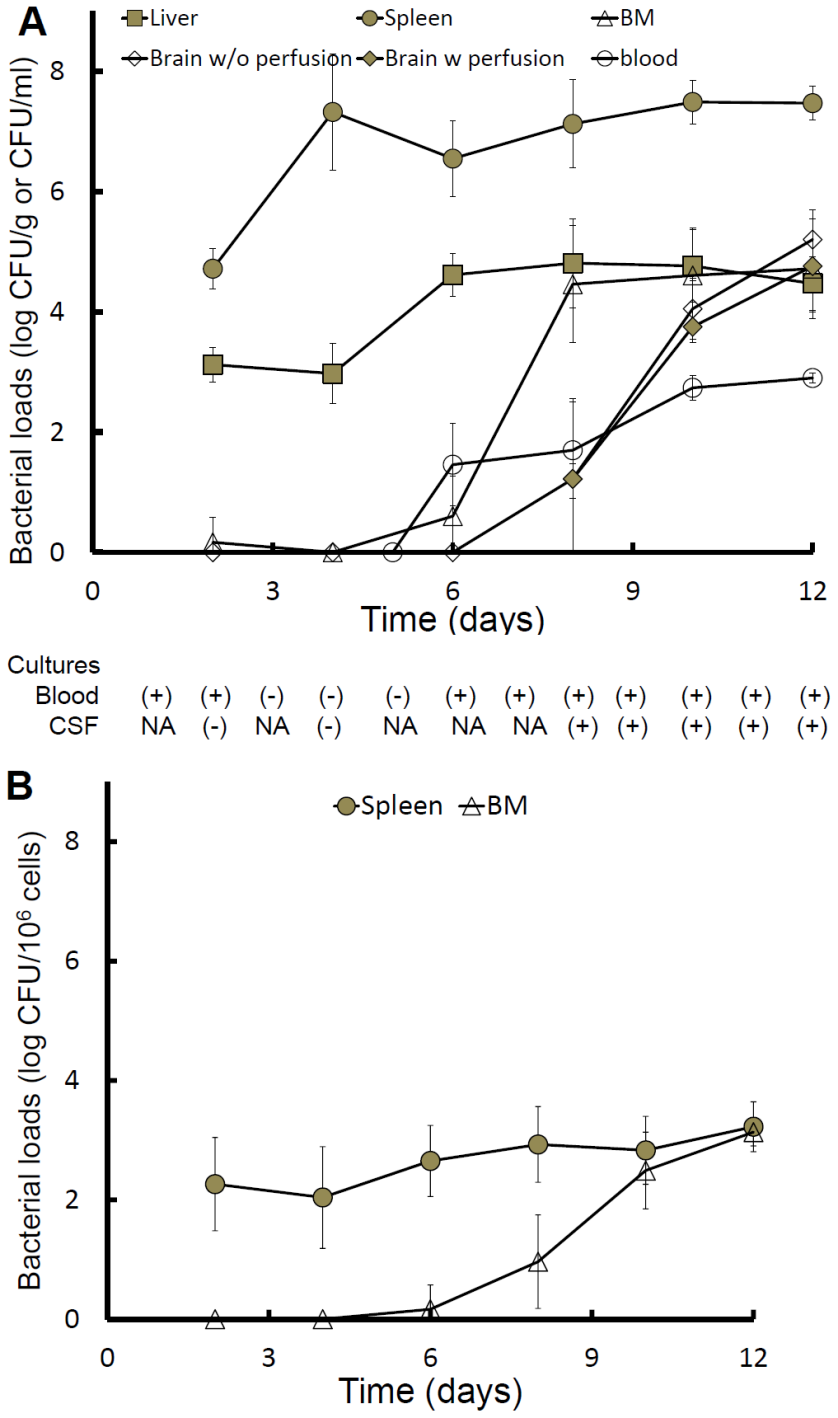
The kinetics of bacterial burdens in organs. At the indicated times, individual mice with melioidosis (n = 6 for each point; independent duplicate experiments) were sacrificed and the numbers of bacteria colonizing the spleen (CFU/g, gray circle), liver (CFU/g, gray square), BM (CFU/ml, hollow triangle), brain without perfusion (CFU/g, hollow diamond) and brain with perfusion (CFU/g, gray diamond) were measured. To determine the bacterial burdens in the blood, 100 µl of blood was directly counted (CFU/ml, hollow circle) using the serial dilution method. Approximately 100 µl of blood or 5 µl of CSF were incubated in Ashdown's broth. The presence (+) or absence (−) of *B. pseudomallei* was observed after a 7-day incubation. NA = not analyzed (A). To measure the amount of intracellular *B. pseudomallei*, single-cell suspensions were prepared from spleens (gray circle) and BM (hollow triangle). The cell suspensions were treated with 400 µg of kanamycin for 2 h, and the number of intracellular bacteria (CFU/10^6^ cells) was subsequently measured by plate counts (B). In this study, the limits of detection were 10, 250 and 3 CFU/g for liver, spleen and brain, respectively, and 10 CFU/ml for both blood and BM. The lower limit of CSF detection was 200 CFU/ml.

The kinetics of bacterial burdens was determined by following a time course. These results indicate that the bacteria colonized the spleen, liver, BM and brain successively and plateaued on days 4 (spleen), 6 (liver), 8 (BM) and 12 (brain) post-infection (Figure 2A). Convincing evidence for substantial bacterial burden in the brain tissue during phase III was obtained by removing intravascular blood and bacteria by perfusion, revealing bacterial burdens of >10^3^ CFU/ml in brain tissues on days 10 and 12 post-infection. Additionally, *B. pseudomallei* was isolated from mouse CSF after 10 d of infection. Bacteremia was not detected by blood cultures on days 3 and 5 post-infection; however, plate counts revealed densities of <10^3^ CFU/ml after 5 d of infection (Figure 2A). Two mice with no neurological signs during phase III simultaneously showed no pathohistological changes in the CNS, negative CSF cultures and negative bacterial loads in the brain (data not shown). We also confirmed that the growth of intracellular bacteria occurred by day 2 in the spleen and by day 6 post-infection in the BM (Figure 2B). Taken together, these data suggest that the primary focus of infection was the spleen (or liver), from which the bacteria subsequently migrated to the BM and later to the brain.

High levels of bacteremia were not capable of inducing the early melioidosis-related meningitis. Neither bacterial brain colonization nor meningeal neutrophil infiltration were observed during day 4 post-infection in mice infected with 5×10^2^, 5×10^3^ or 5×10^4^ CFU of *B. pseudomallei*. All infected mice rapidly died by 4 d of infection with a concentration of ≥5×10^3^ CFU (n = 10, independent duplicate experiments).

### The relationship between CD11b^+^ and Ly6C^+^ population intracellularly infected with *B. pseudomallei* and melioidosis

Because bacterial burdens in the brain were observed after increases in the intracellular bacteria persisting in the spleen and BM, we hypothesized that a vector acting as a Trojan horse that was present in both organs induced melioidosis with CNS symptoms. We first determined the subpopulations of the spleen, BM and PBMCs that carried intracellular *B. pseudomallei*. Prior to this experiment, BALB/c mice were intravenously infected with *B. pseudomallei*-GFP, which was derived from *B. pseudomallei* vgh19. Both GFP and vgh19 strains have identical lethal doses and bacterial burden kinetics in the mouse melioidosis model (see Figure S5; Comparison of survival rates and bacterial burdens in organs from mice intravenously infected with *B. pseudomallei* vgh19 and GFP).

**Figure 3:**
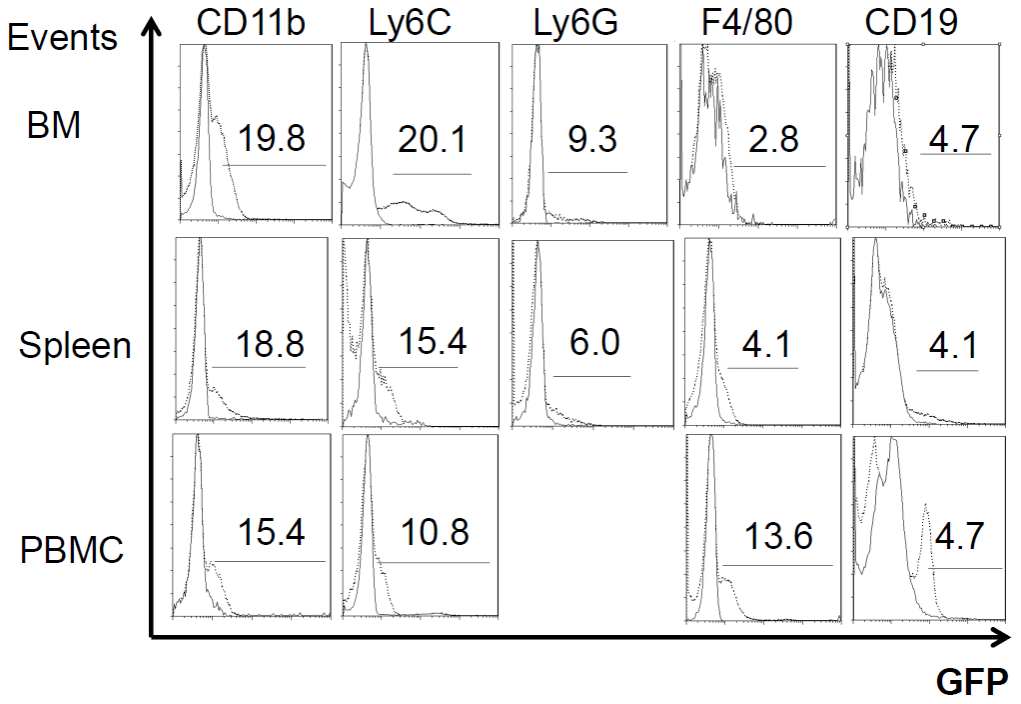
Fluorescent cells in BM, spleen and PBMC subpopulations. Mice were infected with *B. pseudomallei-*GFP. The solid lines represent controls (no infection). The dotted lines represent the fluorescent cells in each phagocytic (CD11b^+^, F4/80^+^, Ly6C^+^ and Ly6G^+^) and lymphoid (CD3^+^, CD4^+^, CD8^+^, CD19^+^ and NK1.1^+^) population on day 10 post-infection. If the GFP was below 1%, the data are not shown.

By analyzing the fluorescence of each population of phagocytic (CD11b^+^, Ly6C^+^, Ly6G^+^ and F4/80^+^) and lymphoid (CD3^+^, CD4^+^, CD8^+^, CD19^+^ and NK1.1^+^) cells (Figure 3, representatives on day 10 post-infection), it was found that both CD11b^+^ and Ly6C^+^ cells were the predominant fluorescent populations in the spleen, BM and PBMC. Distinctly lower amounts of fluorescence were observed in phagocytic Ly6G^+^ and F4/80^+^ cells and lymphoid CD19^+^ cells present in the spleen, BM and PBMC on days 2 and 10 post-infection. Fluorescent CD3^+^, CD4^+^, CD8^+^ and NK1.1 cells were not observed on days 2 and 10 post-infection (Summary in Table 1).

**Table 1:** Summary of the proportion of fluorescent cells in each population.

	Fluorescent cells (%)^a^					
	Spleen		Bone marrow		PBMCs	
	Day 2	Day 10	Day 2	Day 10	Day 2	Day 10
Phagocytic cells						
CD11b	15.5	18.8	1.2	19.8	2.1	15.4
Ly6C	10.3	15.4	1.5	20.1	1.5	10.8
Ly6G	8.2	6.0	#	9.3	#	#
F4/80	2.2	4.1	#	2.8	#	13.6
Lymphoid cells						
CD3	#	#	#	#	#	#
CD4	#	#	#	#	#	#
CD8	#	#	#	#	#	#
CD19	2.2	4.1	#	4.7	1.5	4.7
NK1.1	#	#	#	#	#	#

a The (#) symbol indicates <1% fluorescent cells.

To confirm that *B. pseudomallei* persists in phagocytic or lymphoid cells, the amounts of intracellular bacteria were estimated by isolating splenic or BM CD11b^+^, Ly6C^+^ and CD19^+^ populations and performing plate counts of these populations after serial dilution. There were 1.0–2.23×10^3^ CFU/10^6^ cells in CD11b^+^, 0.43–0.53×10^3^ CFU/10^6^ cells in Ly6C^+^ and 0.06–0.12×10^3^ CFU/10^6^ cells in CD19^+^ populations from the spleen and BM.

### Changes in CD11b^+^ populations during the progression of melioidosis

**Figure 4:**
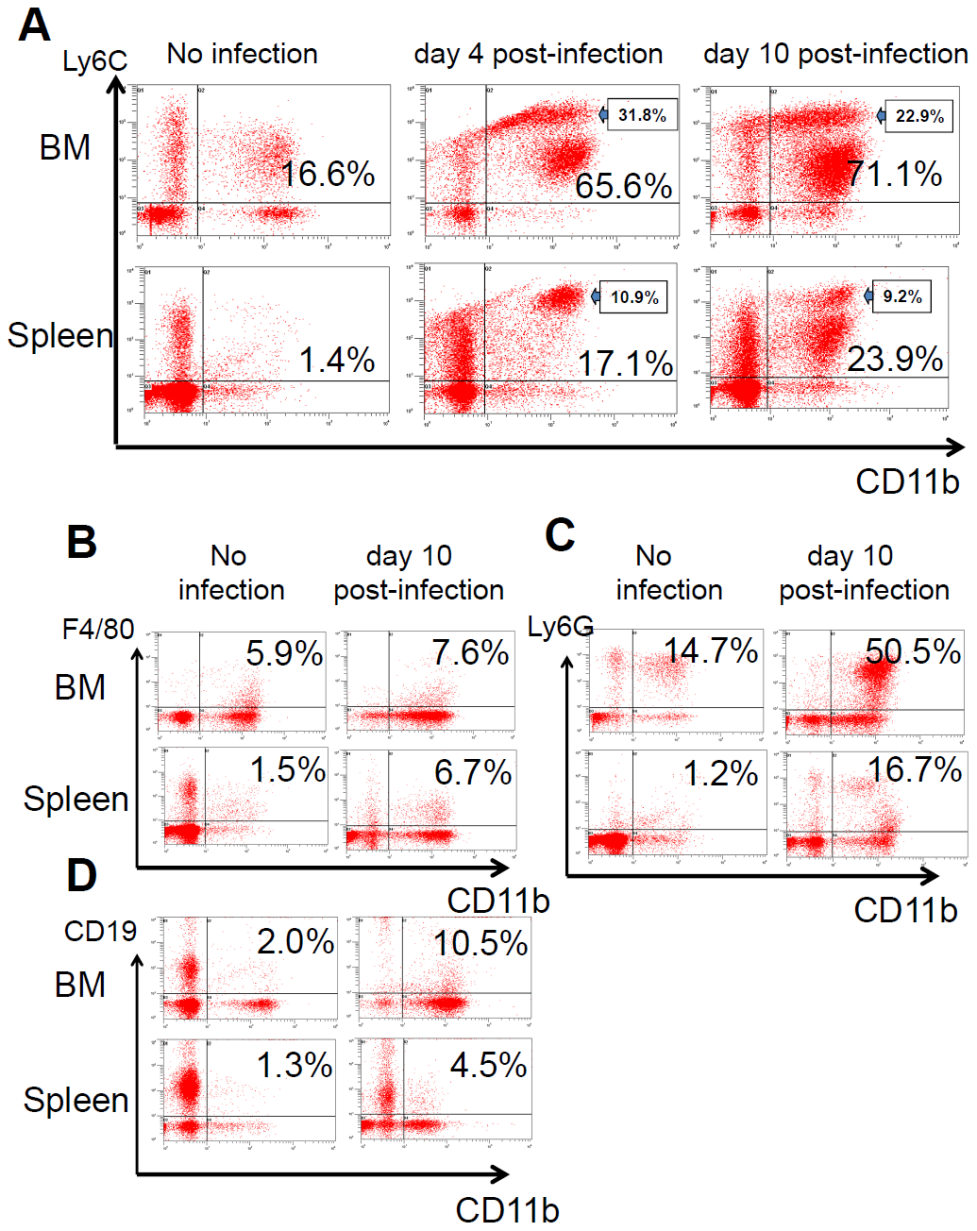
Changes in the CD11b^+^ population during infection. Mice were infected with *B. pseudomallei*-GFP. At the indicated times, the cellular populations were analyzed after double staining for monocytes (A, CD11b^+^Ly6C^+^), macrophages (B, CD11b^+^F4/80^+^), neutrophils (C, CD11b^+^Ly6G^+^) and CD11b^+^CD19^+^ B cells (D).

It is know that the CD11b^+^ population includes inflamed monocytes (CD11b^+^Ly6C^high^), residential monocytes (CD11b^+^Ly6C^low^), neutrophils (CD11b^+^Ly6G^+^), macrophages (CD11b^+^F4/80^+^) and B cells (CD11b^+^CD19^+^). Compared with uninfected mice, the levels of both the CD11b^+^Ly6C^high^ and CD11b^+^Ly6C^low^ cells in the BM of infected mice were increased significantly, reaching 22.9% and 48.2%, respectively (for a total of 71.1%) on day 10 post-infection. In the spleen, the CD11b^+^Ly6C^high^ cells predominated on day 4; however, this predominance shifted to CD11b^+^Ly6C^low^ cells on day 10 post-infection (Figure 4A). In contrast, on day 10 post-infection, macrophage (CD11b^+^F4/80^+^) levels were high in the BM and even higher in the spleens of infected mice compared with those of uninfected mice (Figure 4B). Neutrophils (CD11b^+^Ly6G^+^) were increased by 3.4 and 13.9-fold in the BM and spleen, respectively (Figure 4C), and CD11b^+^CD19^+^ B cells were increased by 5.25 and 3.4-fold in the BM and spleen on day 10 post-infection compared with their levels in uninfected mice (Figure 4D). These results indicate that both phagocytic and lymphoid CD11b^+^ cells in the BM and spleen were expanded on day 10 post-infection.

### Induction of bacterial burdens in brains by adoptive transfer of infected cells

**Figure 5:**
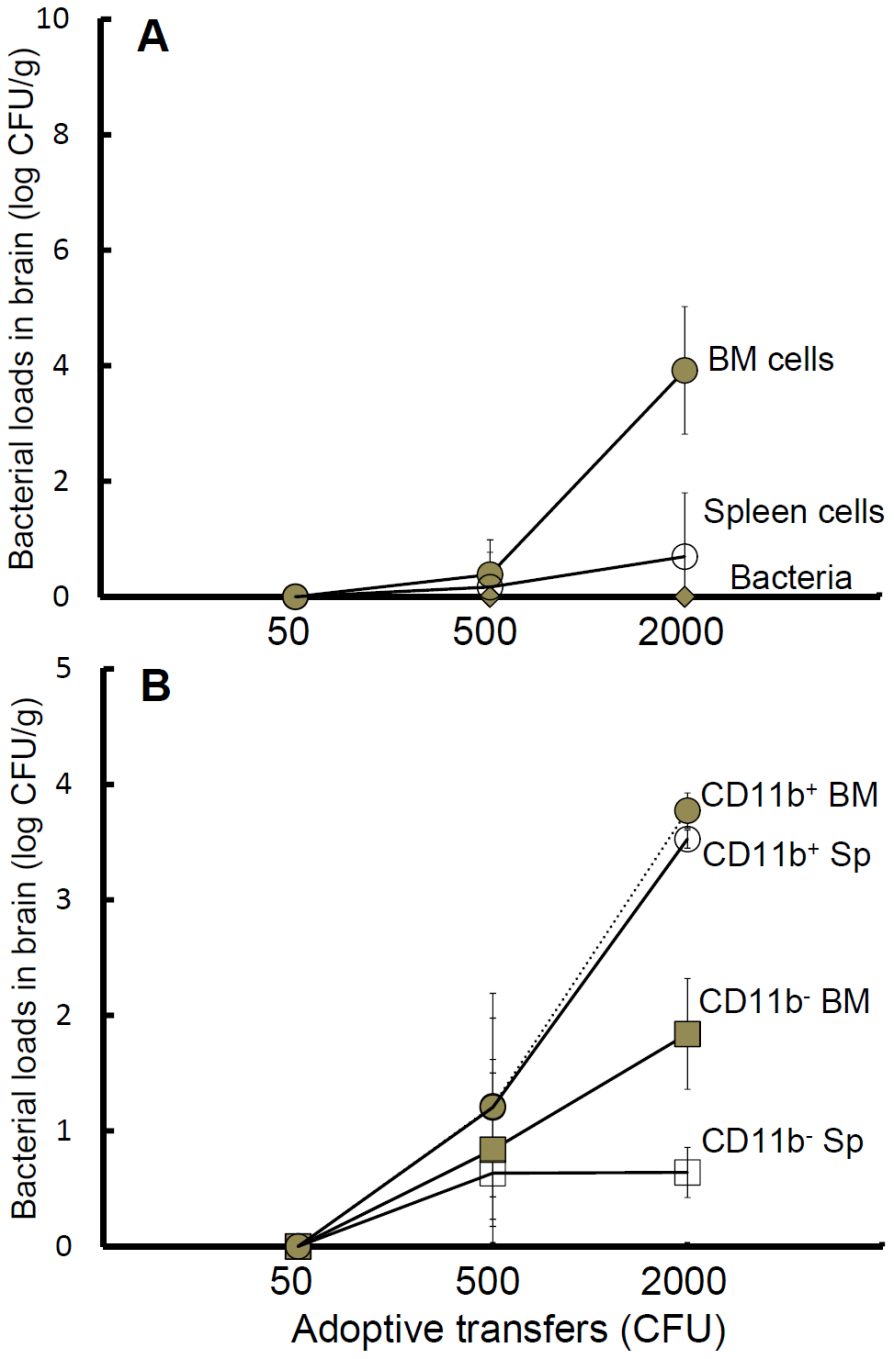
Adoptive transfers of infected cells. On day 10 post-infection, splenic (hollow circle) and BM (gray circle) cells were isolated and were adjusted to normalize the numbers (50, 500 or 2000 CFU) of intracellular *B. pseudomallei*. Extracellular (free) *B. pseudomallei* (50, 500 or 2000 CFU; gray diamond) were obtained from destroyed BM cells (A). Simultaneously, CD11b^+^ and CD11b^−^ cells were isolated from the spleen (hollow circle, CD11b^+^; hollow square, CD11b^−^) and BM (gray circle, CD11b^+^; gray square, CD11b^−^) (B). Two days after adoptive transfer, bacterial burdens (CFU/g) in the brain were determined in the recipients (n = 3, independent duplicate experiments). The limits of detection were 3 CFU/g for brain tissue.

To determine whether the induction of bacterial infection in the brain was due to an infected cell used as a “Trojan horse”, the infected BM and splenic cells were isolated and adoptively transferred to healthy individuals. In this study, no adverse responses (deaths, signs of illness such as shivering or decreased activity; or pathohistological changes in the spleen, liver and brain) were observed after adoptive transfer of uninfected donor cells (10^4^–10^6^ cells) (data not shown). However, adoptive transfer of either infected BM or splenic cells increased the amount of brain-colonizing bacteria in a dose-dependent manner (Figure 5A). As in the above studies, we have demonstrated that the proportion of the CD11b^+^ population in the BM was higher than that in the spleen (Figure 4). Thus, we isolated CD11b^+^ and CD11b^−^ populations from both organs and adjusted the isolated cells to obtain equal numbers of intracellular bacteria (50, 500 or 2000 CFU). Two days after adoptive transfer with CD11b^+^ cells from either the BM or spleen, significant bacterial burdens were observed in brains of the recipient mice (Figure 5B).

We demonstrated that extracellular bacteria doses (free *B. pseudomallei*) of 50 to 5000 CFU prepared from LB cultures were not able to cause bacterial infection in the brains by day 4 post-infection as described above. To rule out the possibility that the increased ability of the bacteria to colonize the brain is due to host adaptation in the donor mice, the infecting *B. pseudomallei* (50, 500 or 2000 CFU) were harvested from disrupted BM or splenic cells and subsequently injected intravenously. On day 2 post-injection, no colonizing bacteria were found in the brain (Figure 5A). However, the survival rates and the bacterial burdens were identical between mice infected with extracellular *B. pseudomallei* obtained from disrupted cells and the mice infected with *B. pseudomallei* from LB cultures (data not shown). The invasive ability of extracellular *B. pseudomallei* was not improved after adaptation in the host.

### Association between CD11b^+^ selectin-expressing cells and bacterial burdens in brain

**Figure 6:**
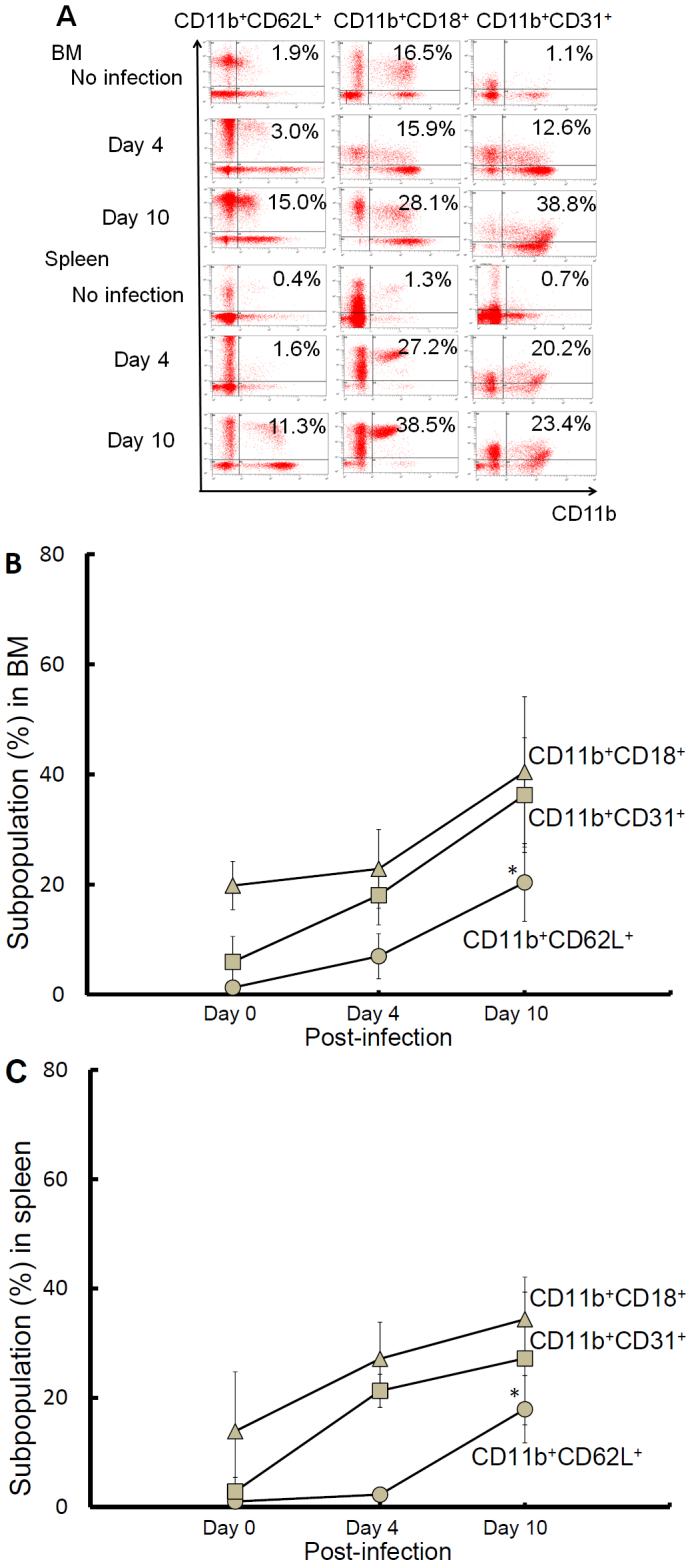
Changes in CD11b^+^ selectin- and integrin-expressing cells during melioidosis. At the indicated times, splenic or BM cells were isolated and stained with fluorescence-conjugated anti-CD11b and CD62L (or CD18 or CD31) antibodies. As a representative sample, the proportion of CD11b^+^ selectin (CD62L))- or integrin (CD18 or CD31)-expressing cells is shown (A). The average changes in the CD11b^+^CD62L^+^ (gray circle), CD11b^+^CD18^+^ (gray triangle) and CD11b^+^CD31^+^ (gray square) populations (independent mice, n = 3; independent duplicate experiments) are shown for BM (B) and spleen (C). The (*) symbol indicates *p*<0.05 (day 4 vs. day 10).

Because we found that CD11b^+^ cells carrying intracellular *B. pseudomallei* play a role in inducing bacterial colonization of the brain, we next measured the expression of selectin (CD62L) and integrin (CD18 or CD31) on CD11b^+^ cells during the progression of melioidosis because these molecules are involved in the transmigration of leukocytes. Our results revealed that the CD11b^+^CD62L^+^, CD11b^+^CD31^+^ and CD11b^+^CD18^+^ populations were substantially increased after 10 d of infection (Figure 6A, for representative data). Of the populations that varied between days 4 and 10 post-infection, both CD11b^+^CD31^+^ and CD11b^+^CD18^+^ populations showed mild increases ranging from 1.15 to 3-fold in the BM (Figure 6B) and spleen (Figure 6C); however, CD11b^+^CD62L^+^ population increased as much as 7.1-fold in the spleen (Figure 6C).

**Figure 7:**
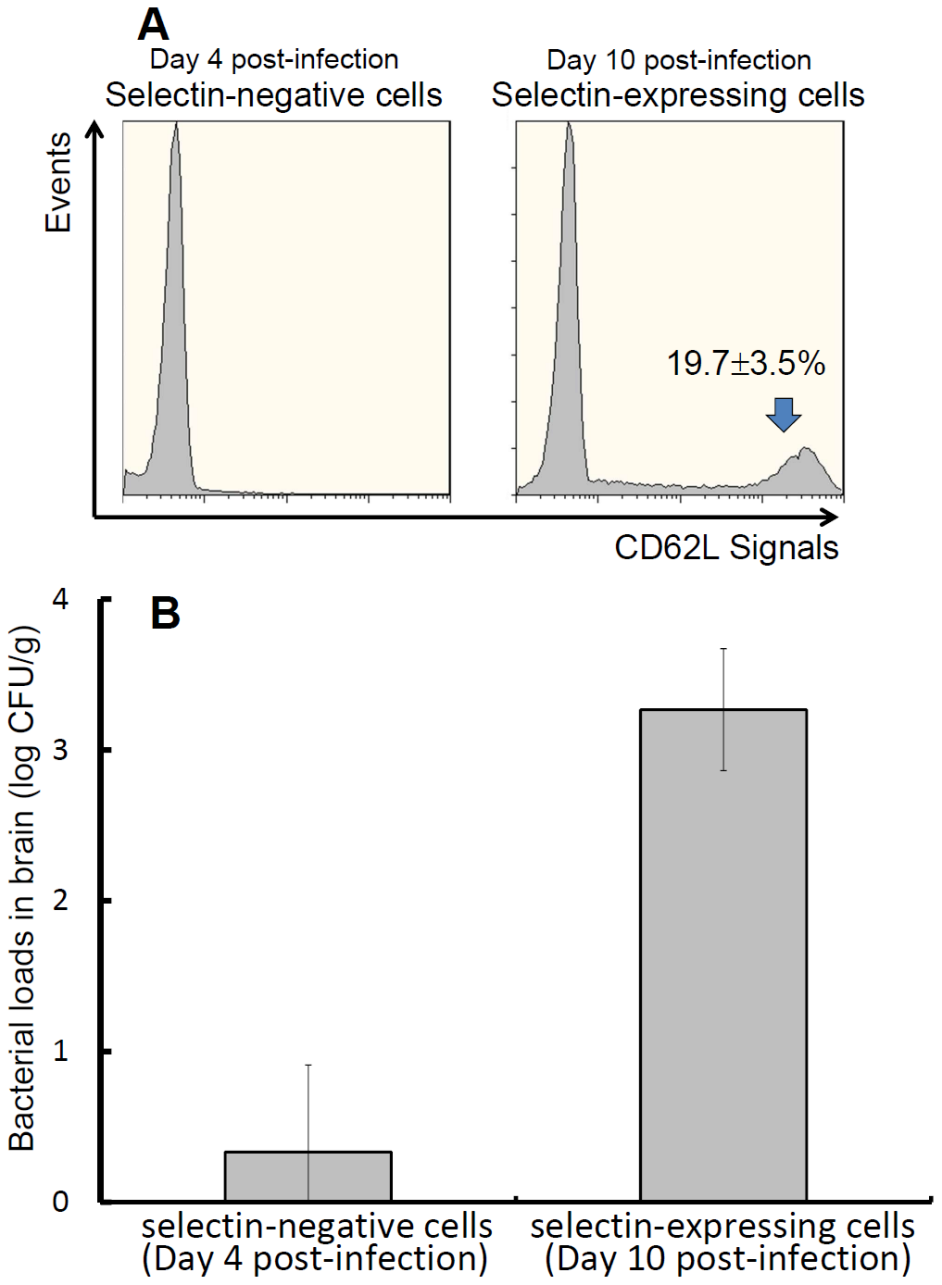
Expression and adoptive transfer of CD11b^+^ selectin-expressing cells. Splenic CD11b^+^ cells were isolated from mice with melioidosis on days 4 or 10 post-infection. The proportions of CD11b^+^ selectin-negative cells (left) and selectin-expressing cells (right) were confirmed (A). The CD11b^+^ cells were obtained from the same mice described above on day 4 (selectin-negative cells) and day 10 (selectin-expressing cells), and adoptive transfer was performed. After 2 d, the bacterial burdens (CFU/g) in the brains of the recipients were determined (B). The values in all experiments are presented as the mean ± SD of 3 independent duplicate experiments. The limit of detection was 3 CFU/g.

On day 4 post-infection, bacteria were not observed in the brain (Figure 2A); however, intracellular *B. pseudomallei* has persisted in the spleen (Figure 2B). Besides, the splenic CD11b^+^ population increased on day 4 post-infection (Figure 4A) and CD11b^+^ cells harboring *B. pseudomallei* isolated from the spleen (on day 10 post-infection) induced bacterial brain colonization (Figure 5B). Thus, we hypothesized that increased selectin expression on infected splenic cells is related to an increased bacterial burden in brain. We demonstrated that a majority of the splenic CD11b^+^ population lacked surface CD62L (defined as selectin-negative cells) on day 4 post-infection. However, the proportion of selectin-expressing cells increased to 19.7±3.5% on day 10 post-infection (Figure 7A). The bacterial burdens in the brains of recipients were rapidly increased with the adoptive transfer of CD11b^+^ selectin-expressing cells but not CD11b^+^ selectin-negative cells (Figure 7B). This result suggests that surface expressed L-selectin may be an important molecule involved in the development of *B. pseudomallei* brain infection.

### The occurrence of melioidosis with meningitis after adoptive transfer

**Figure 8:**
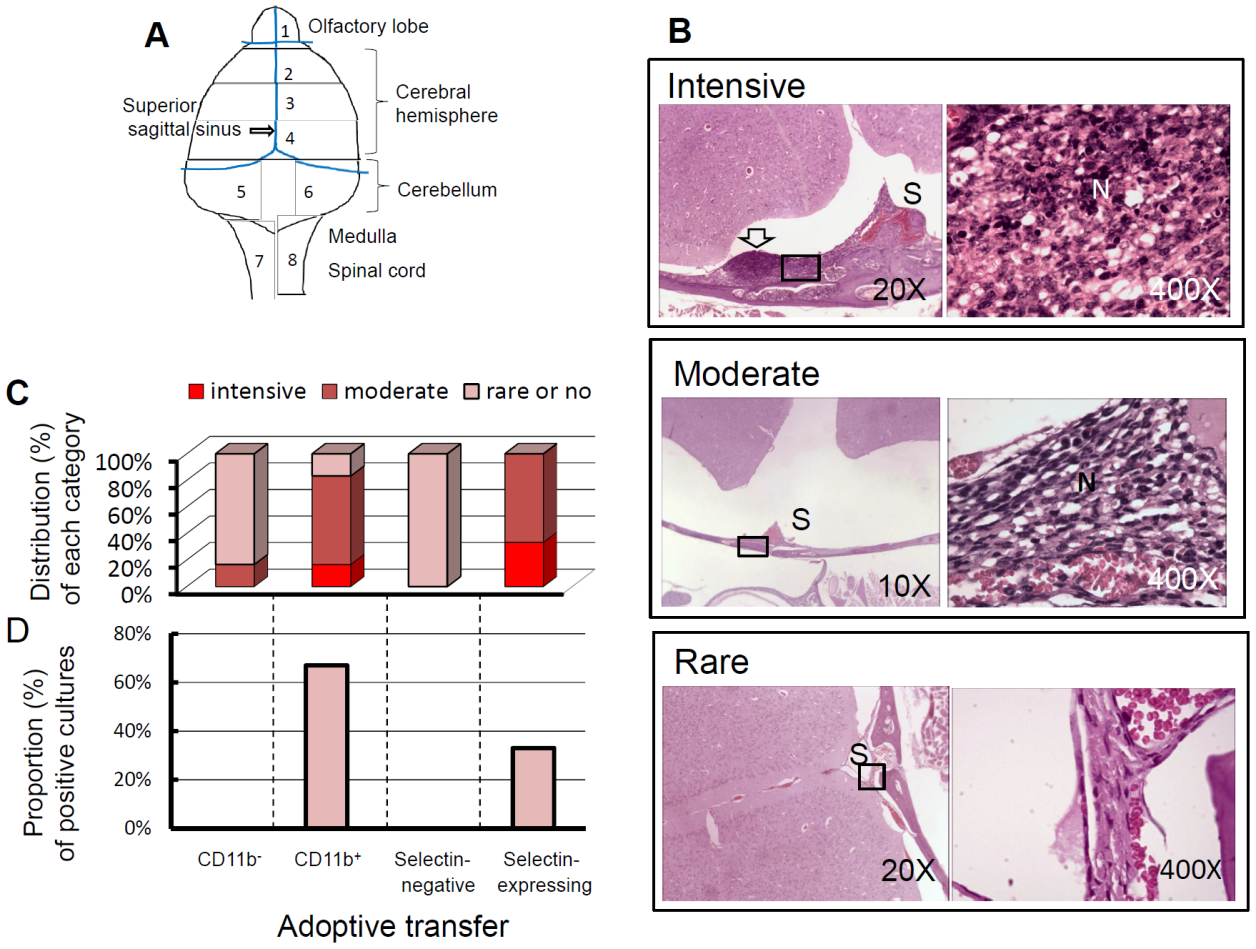
Histological and bacteriological examination of the brains of recipients after adoptive transfer. The mice were sacrificed (n = 3, independent duplicate experiments) on day 2 post-transfer. A total of 8 wax blocks were prepared as shown. Histological changes in the meninges were observed only in blocks 2 and 3 (A). Representative images of intensive and moderate meningeal neutrophil infiltration and vascular congestion with rare cell infiltration are shown (10× or 20×, left column; 400×, right column, magnified from the black square). The position of the superior sagittal sinus (S) and the presence of neutrophils (N) or abscesses (arrow) are indicated (B). The distribution (%) of each histological category (intensive, moderate and rare neutrophil infiltration) is shown (C). Simultaneously, CSF samples were collected and seeded into culture bottles. The proportion (100%) of positive *B. pseudomallei* cultures obtained from the tested mice is shown. The lower limit of CSF detection was 200 CFU/ml (D).

After adoptive transfer of splenic CD11b^+^ or selecting-expressing cells (harboring 2000 CFU of cultivated *B. pseudomallei*), mouse melioidosis rapidly progressed (Table S4, Summary of the characteristics of melioidosis progression after adoptive transfer). Both histological and bacteriological analyses were performed to evaluate the development of melioidosis-associated meningitis (n = 3, each analysis was performed in duplicate). For histological sectioning, tissue blocks from each mouse were prepared in the same way (Figure 8A, positioning of the tissue blocks). The degrees of meningeal inflammation were determined based on neutrophil infiltration (i.e., intensive, moderate or rare infiltration) (Figure 8B, for representative histological images). In the mouse groups that received adoptively transferred infected splenic CD11b^+^ or selectin-expressing cells, the intensive neutrophil infiltration (17%, for the adoptively transferred group of CD11b^+^ cells; 33%, for the transferred group of selectin-expressing cells) and moderate neutrophil infiltration (67%, for both groups of CD11b^+^ and selectin-expressing cells) were observed in brain tissue (Figure 8C). The major foci were localized in the regions surrounding cerebral superior sagittal sinus for all infected mice, whereas brain abscesses and brain stem encephalitis were not observed. The observation of meningeal neutrophil infiltration with cellular necrosis agreed that bacterial burdens in the brains were rapidly increased after adoptive transfer of CD11b^+^ cells (Figure 5B) or selectin-expressing cells (Figures 7B). In contrast, in the mouse groups that received adoptively transferred infected splenic CD11b^−^ cells or selectin-negative cells, the bacterial colonization of the brain was absent or rare (Figure 5B, 7B). The typical neutrophil infiltration into the meninges was also not observed in these mice, although vascular congestion with a few cells surrounding the blood vessels was noted (Figure 8C). The induction of melioidosis with meningitis was reconfirmed based on the growth of *B. pseudomallei* in the CSF of mice that received adoptively transferred infected CD11b^+^ or selectin-expressing cells. However, *B. pseudomallei* was never isolated from the CSF of mice that received adoptively transferred infected CD11b^−^ or selectin-negative cells on day 2 post-transfer (Figure 8D).

## Discussion

The BALB/c mouse, a model that reflects the clinical course of human acute melioidosis [3], [4], [6], [10], [19], [20], was used to obtain reproducible manifestations of neurological melioidosis that includes aspects of leptomeningitis, meningoencephalitis, encephalomyelitis and brain abscesses secondary to remote infected foci. After adoptive transfer of infected cells, the initial meningeal foci were localized in the regions surrounding cerebral superior sagittal sinus because, on day 2 post-transfer, macroscopic brain abscesses, encephalomyelitis, brain stem encephalitis and even neutrophil infiltration in another meningeal place were not found. It has been suggested that *B. pseudomallei* infection in the brain is caused by bacteria traveling from the olfactory nerve to the brain, resulting in CNS symptoms after the initial intranasal infection [39]. However, inhalation, ingestion and subcutaneous inoculation, which were the possible melioidosis infection routes, subsequently resulted in systemic dissemination. Although bacterial colonization in the brain, neutrophil infiltration in the meninges and bacterial cultures from the CSF were characterized by intravenous infection in this study, the mechanisms of developing a CNS infection most likely differ if the infection was initiated through different means. Meningitis may be frequently overlooked because it is secondary to the primary hematogenous spread of the melioidosis associated with acute sepsis [15]. This study is the first analysis of the manifestations of melioidosis-associated meningitis in a mouse model.

Primary meningitis-causing bacteria such as *Escherichia coli*, group B *Streptococcus* and *Neisseria meningitides* commonly trigger translocation across the vascular endothelium *via* the binding of a ligand to a specific receptor [40]–[42]. Aside from this mechanism, it has been reported that monocytic Ly6C^+^ cells shelter intracellular *Listeria monocytogenes*. These cells, in a so-called Trojan horse mechanism, act as a vessel to allow the bacteria to cross into the brain [43]–[45]. In this study, melioidosis with meningitis exhibited the following characteristics: (1) Severe bacteremia was not necessary; (2) neutrophil infiltration into the meninges and bacterial burdens in the brain occurred during the second (phase III) but not the first (phase I) decline phase; (3) meningitis was induced by infected CD11b^+^ cells but not by extracellular (free) bacteria; and (4) selectin was involved in the onset of meningitis. These results imply that *B. pseudomallei*-infected cells likely act as a Trojan horse, an important cause of melioidosis with meningitis.

After primary *B. pseudomallei* infection, splenitis and hepatitis, including necrotic areas composed of degraded and viable macrophages and neutrophils, are commonly observed [10], [31], [37]. It has been reported that mice with fulminating melioidosis produce high levels of TNF-α, IL-6, IFN-γ and MCP-1, whereas infected but asymptomatic mice do not [6], [19], [46]. Our results revealed that, on day 5 post-infection, these inflammatory cytokines were significantly decreased in the serum; however, the expression of the macrophage activation- and attraction-related cytokines IFN-γ and MCP-1 increased on day 10 post-infection. Simultaneously, neutrophil/monocyte infiltration into the meninges was observed. This result supports previous reports indicating that MCP-1 mediates the recruitment of CCR2^+^ monocytes to the inflamed CNS [45], [47]. However, TNF-α and IL-6 play essential roles in the control of systemic *B. pseudomallei* infection [48], [49]. Additionally, IL-12 is essential for the production of IFN-γ. IFN-γ and/or bacterial lipopolysaccharide (LPS) are ligands known to induce the activation of highly microbicidal macrophages (M1) [50]. In this study, the lack of TNF-α, IL-6 and IL-12 induction on day 10 post-infection could result in an incomplete immune response against *B. pseudomallei*. Consequently, the growth of the intracellular bacteria was not restricted; instead, they burst their carrier cells and colonized the brain.

The timing of bacterial colonization in the spleen and BM was related to the expansion of splenic and BM CD11b^+^ cells, a predominant population that carried more intracellular *B. pseudomallei* than BM Ly6C^+^ and BM CD19^+^ cells. This result implies that *B. pseudomallei* exhibits different invasive tropisms toward phagocytic and lymphoid cells. *B. pseudomallei* is capable of persisting in neutrophils, macrophages and/or monocytes during early infection based on the presence of fluorescent cells in splenic CD11b^+^, Ly6C^+^, Ly6G^+^ and F4/80^+^ populations on day 2 post-infection. CD11b, a subunit of the adhesion molecule of Mac-1 (CD11b/CD18, α_M_β_2_, CR3), is highly expressed on antigen-presenting cells, monocytes, neutrophils and B cells and is involved in the transmigration of these cells [51]–[53]. Generally, CD11b^+^ neutrophils are significantly expanded during gram-negative bacterial infection compared with gram-positive bacterial infection [54]. We have demonstrated that neutrophils (CD11b^+^Ly6G^+^) and monocytes (CD11b^+^Ly6C^high^ and CD11b^+^Ly6C^low^) are expanded in the BM and spleen during melioidosis. It is not clear how the initial inflammatory events in the brain occur; however, monocytes and neutrophils are increased in inflamed tissues in mice with melioidosis [19], [22], [23]. Thus, we suggest that the *B. pseudomallei*-loaded CD11b^+^ cells provided the factor necessary for the systemic dissemination of the infection *via* the bloodstream and the subsequent breach of the endothelial cells to infect the brain.

The Ly6C^high^ population (inflamed monocytes) becomes expanded by infection with *L. monocytogenes* in the BM. All the *L. monocytogenes*-infected cells were CD11b^+^ monocytes (approximately 90% of the CD11b^+^ population simultaneously expressed Ly6C in the cases of listeriosis) [45]. These infected cells were released from the BM into the circulation and ultimately invaded the CNS [45], [55]–[57]. In this study, by following a bacterial burden time course, it appeared likely that the infected cells that induced melioidosis-associated meningitis originated from the BM. However, we found that *B. pseudomallei* persisted intracellularly in phagocytic and lymphoid cells. Indeed, adoptive transfer of infected BM CD11b^−^ cells (most likely CD11b^−^Ly6C^+^ or CD11b^−^CD19^+^ cells) resulted in some degree of bacterial brain colonization. Additionally, lymphocytes were accumulated in the meninges based on histological examination. Moreover, it has been reported that *B. pseudomallei* enters lymphocytes and replicates intracellularly [58]. Thus, we suggest that all infected cells from the spleen and BM could simultaneously or synergistically contribute to meningitis.

In accordance with the Trojan horse mechanism, *B. pseudomallei* is shuttled to the brain within immune cells [34], [41], [59]. The cell adhesion molecules selectin (CD62L) and integrin (CD18 and CD31), expressed on CD11b^+^ cells, facilitate transmigration through the endothelial cells, and increases in the levels of these adhesion molecules facilitate the development of meningitis. In this study, mouse melioidosis with meningitis was induced by a low bacterial dose (50 CFU); however, compared with other organs, the spleen is a more permissive environment for bacterial replication in mice infected with lethal, sublethal or very low doses [10], [31]. We propose that splenic CD11b^+^ cells act as reservoirs, harboring intracellular *B. pseudomallei* during early infection. CD11b^+^Ly6C^+^CD62L^+^ cells are inflammatory, whereas CD11b^+^Ly6C^−^CD62L^−^ cells are residential monocytes in the mouse spleen [60], [61]. Exacerbated melioidosis could develop in concert with meningitis when splenic inflammatory CD11b^+^ selectin-expressing cells gradually increase during disease progression.

## Supporting Information

Figure S1 Construction of the conjugative plasmid. Wild type and truncated *wbiI* encoding 639 and 365 amino acids, respectively, are shown (A). The conjugative plasmid (pKNOCK *wbiI*-p*fliC*-*gfp*-*cat*) containing truncated *wbiI* was integrated into chromosome 1 of *B. pseudomallei* vgh19. After recombination, a functional *wbiI* and a defective *wbiI* were generated (B).
(TIF)

Figure S2 Scheme of the adoptive transfer protocols. Refer to Text S1 for the protocols (step 1–10). (A) After isolating donor cells (a representative CD11b^+^ population) from infected mice (on days 4 or 10 post-infection), three preliminary experiments, including fluorescence microscopic observation (Exp 1), determination of fluorescent (F) cells by flow cytometric analysis (Exp 2) and plate counts after serial dilution (Exp 3), were respectively performed. (B) The numbers of viable *B. pseudomallei* to 1000 of intracellular GFP cells were shown. Both spleen (Sp) and bone marrow (BM) cells were isolated from the mice on day 10 post-infection. The CD11b^+^ selectin-negative (CD62L^−^) and selectin-expressing (CD62L^+^) cells were prepared from mouse spleens on days 4 and 10 post-infection. Using isolation kits (STEMCELL Tech), BM CD11b^+^ and CD11b^−^ cells as well as spleen CD11b^+^ and CD11b^−^ cells were isolated from mice on day 10 post-infection. Means ± SD were derived from duplicate experiments, each involving 3 independent mice per condition. (C) For adoptive transfer, the donor cells were adjusted to carry 50, 500 or 2000 CFU in accordance with the above preliminary data (i.e., the ratio of viable *B. pseudomallei* to fluorescent cells). The data were unavailable if intracellular *B. pseudomallei* within the donor cells used for adoptive transfer were determined to be >±15% of 50, 500 or 2000 CFU by plate counts.
(TIF)

Figure S3 The survival rates of mice with melioidosis. Survival rates (100%) were recorded daily for BALB/c mice (n = 20, each group) infected with high (500 CFU, gray square), intermediate (50 CFU, black square) and low (5 CFU, white square) doses of *B. pseudomallei* vgh19.
(TIF)

Figure S4 Cytokines and liver function enzymes in mouse sera. The mice were intravenously infected with *B. pseudomallei* (50 CFU). On days 0, 2, 5 and 10 post-infection, the serum cytokines IFN-γ (gray square), IL-6 (gray diamond), IL-10 (gray triangle), IL-12 (star), TNF-α (hollow circle) and chemokine (MCP-1, gray circle) (A) as well as the serum enzymes GOT (gray circle) and GPT (gray square) (B) were measured. The means ± SD were derived from 6 independent mice in duplicate experiments. The (*) symbol indicates *p*<0.05 compared with the data derived from day 5 post-infection.
(TIF)

Figure S5 Comparison of survival rates and bacterial burdens in organs from mice intravenously infected with *B. pseudomallei* vgh19 and GFP. Survival rates (100%) were recorded on the indicated day for BALB/c mice (n = 10, each group) infected with *B. pseudomallei* vgh19 (50 CFU, dark gray) or GFP (50 CFU, light gray) (A). At the indicated times, individual mice with melioidosis (each experiment, n = 6; duplicate experiments) were sacrificed, and their bacterial burdens in the liver (B) and spleen (C) were determined. The limits of detection were 10 and 250 CFU/g for the liver and spleen, respectively.
(TIF)

Table S1 Clinical score parameters, assessed values and weight score. (DOC)

Table S2 Primary antibodies used for flow cytometry in this study. (DOC)

Table S3 Summary of the characteristics of melioidosis progression during intravenous infection. (DOC)

Table S4 Summary of the characteristics of melioidosis progression after adoptive transfer. (DOC)

Text S1 Protocols and comments of adjusting donor cells. (DOC)
