# Supplementary figures and images for: Induction of Mouse Melioidosis with Meningitis by CD11b+ Phagocytic Cells Harboring Intracellular B. pseudomallei as a Trojan Horse
Source: PLoS Negl Trop Dis. 2013 Aug 8;7(8):e2363. doi: 10.1371/journal.pntd.0002363 (PMC3738478; doi:10.1371/journal.pntd.0002363)

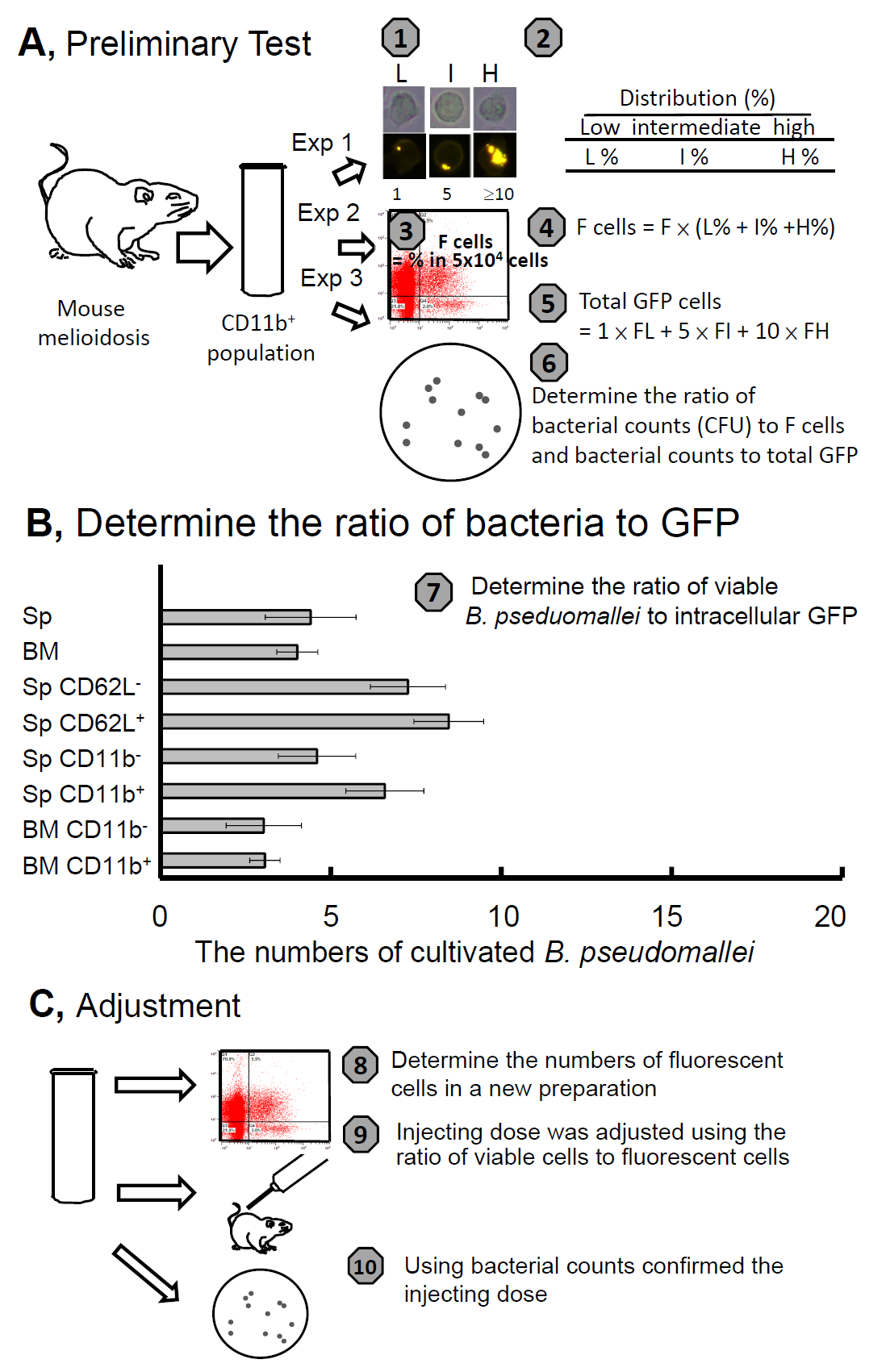

Supplement: Figure S2 — Refer to Text S1 for the protocols (step 1–10). (A) After isolating donor cells (a representative CD11b+ population) from infected mice (on days 4 or 10 post-infection), three preliminary experiments, including fluorescence microscopic observation (Exp 1), determination of fluorescent (F) cells by flow cytometric analysis (Exp 2) and plate counts after serial dilution (Exp 3), were respectively performed. (B) The numbers of viable B. pseudomallei to 1000 of intracellular GFP cells were shown. Both spleen (Sp) and bone marrow (BM) cells were isolated from the mice on day 10 post-infection. The CD11b+ selectin-negative (CD62L−) and selectin-expressing (CD62L+) cells were prepared from mouse spleens on days 4 and 10 post-infection. Using isolation kits (STEMCELL Tech), BM CD11b+ and CD11b− cells as well as spleen CD11b+ and CD11b− cells were isolated from mice on day 10 post-infection. Means ± SD were derived from duplicate experiments, each involving 3 independent mice per condition. (C) For adoptive transfer, the donor cells were adjusted to carry 50, 500 or 2000 CFU in accordance with the above preliminary data (i.e., the ratio of viable B. pseudomallei to fluorescent cells). The data were unavailable if intracellular B. pseudomallei within the donor cells used for adoptive transfer were determined to be >±15% of 50, 500 or 2000 CFU by plate counts. (TIF) [file pntd.0002363.s002.tif]

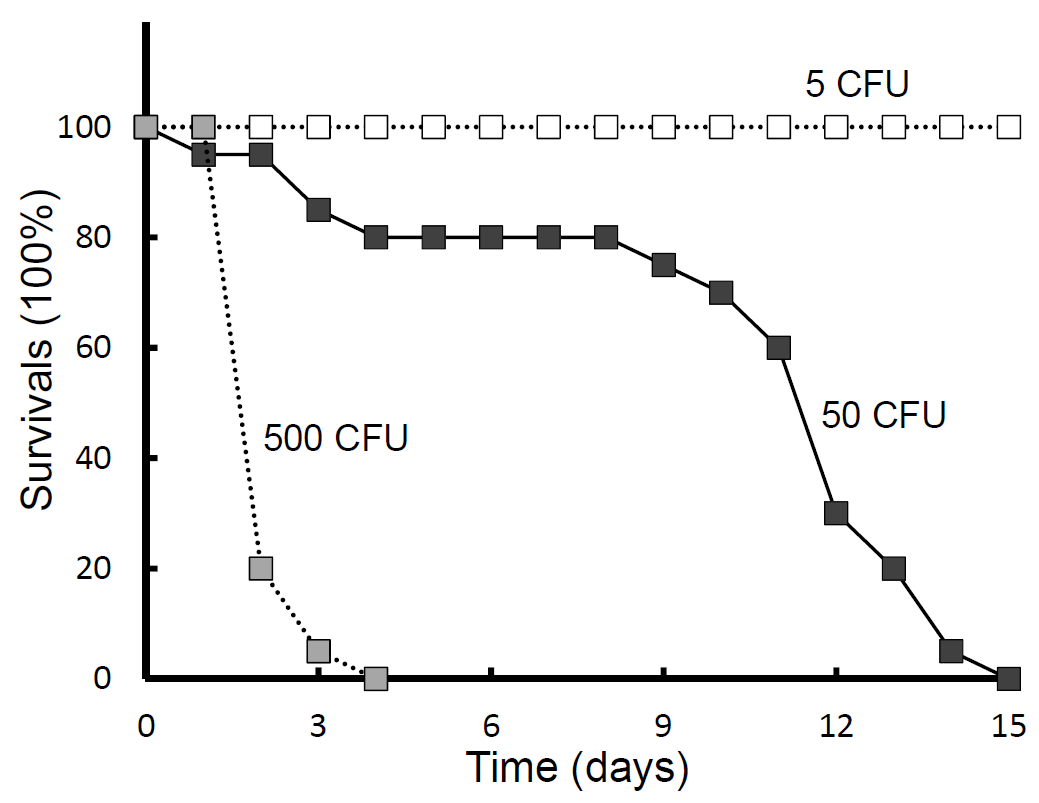

Supplement: Figure S3 — Survival rates (100%) were recorded daily for BALB/c mice (n = 20, each group) infected with high (500 CFU, gray square), intermediate (50 CFU, black square) and low (5 CFU, white square) doses of B. pseudomallei vgh19. (TIF) [file pntd.0002363.s003.tif]

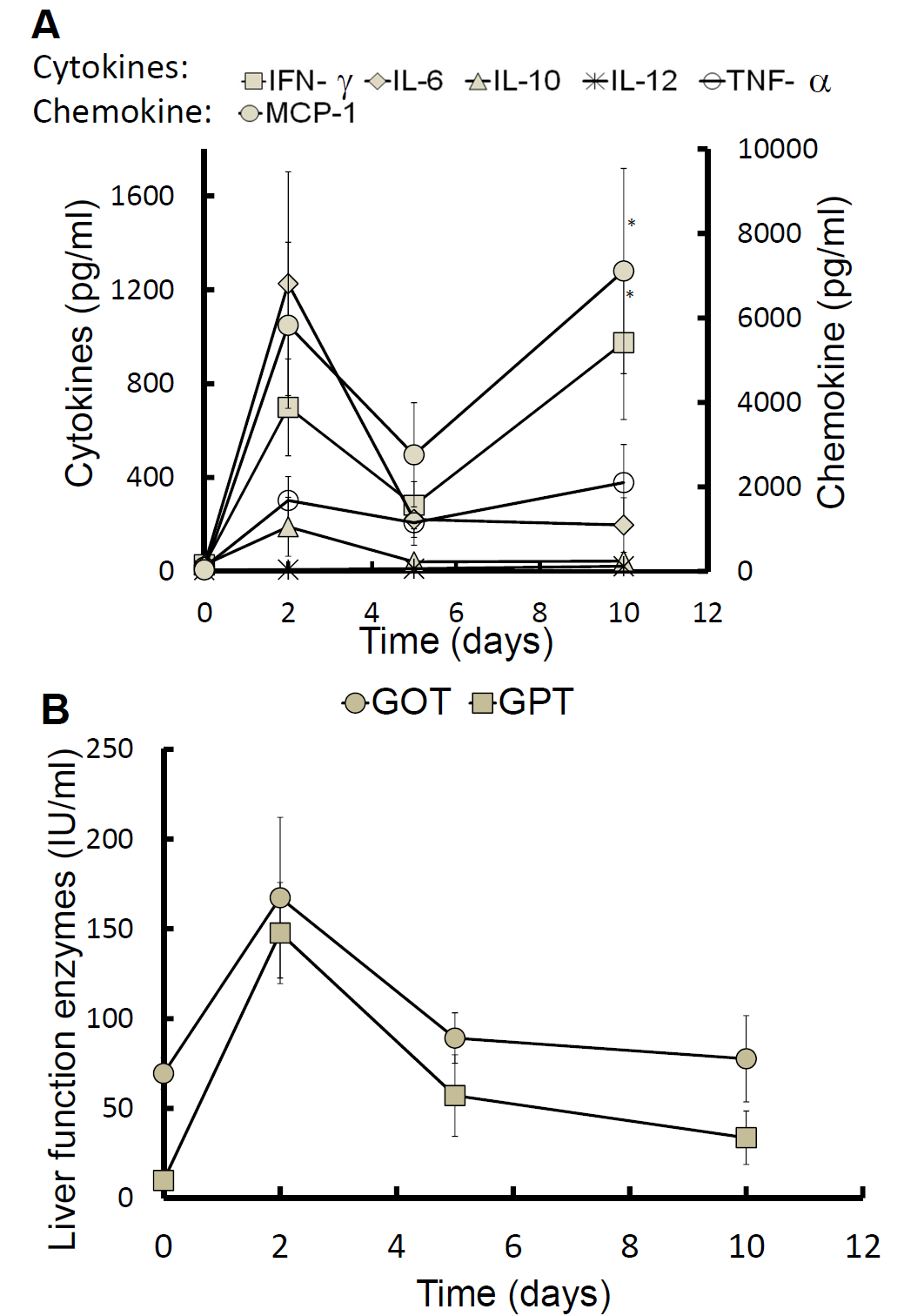

Supplement: Figure S4 — The mice were intravenously infected with B. pseudomallei (50 CFU). On days 0, 2, 5 and 10 post-infection, the serum cytokines IFN-γ (gray square), IL-6 (gray diamond), IL-10 (gray triangle), IL-12 (star), TNF-α (hollow circle) and chemokine (MCP-1, gray circle) (A) as well as the serum enzymes GOT (gray circle) and GPT (gray square) (B) were measured. The means ± SD were derived from 6 independent mice in duplicate experiments. The (*) symbol indicates p<0.05 compared with the data derived from day 5 post-infection. (TIF) [file pntd.0002363.s004.tif]

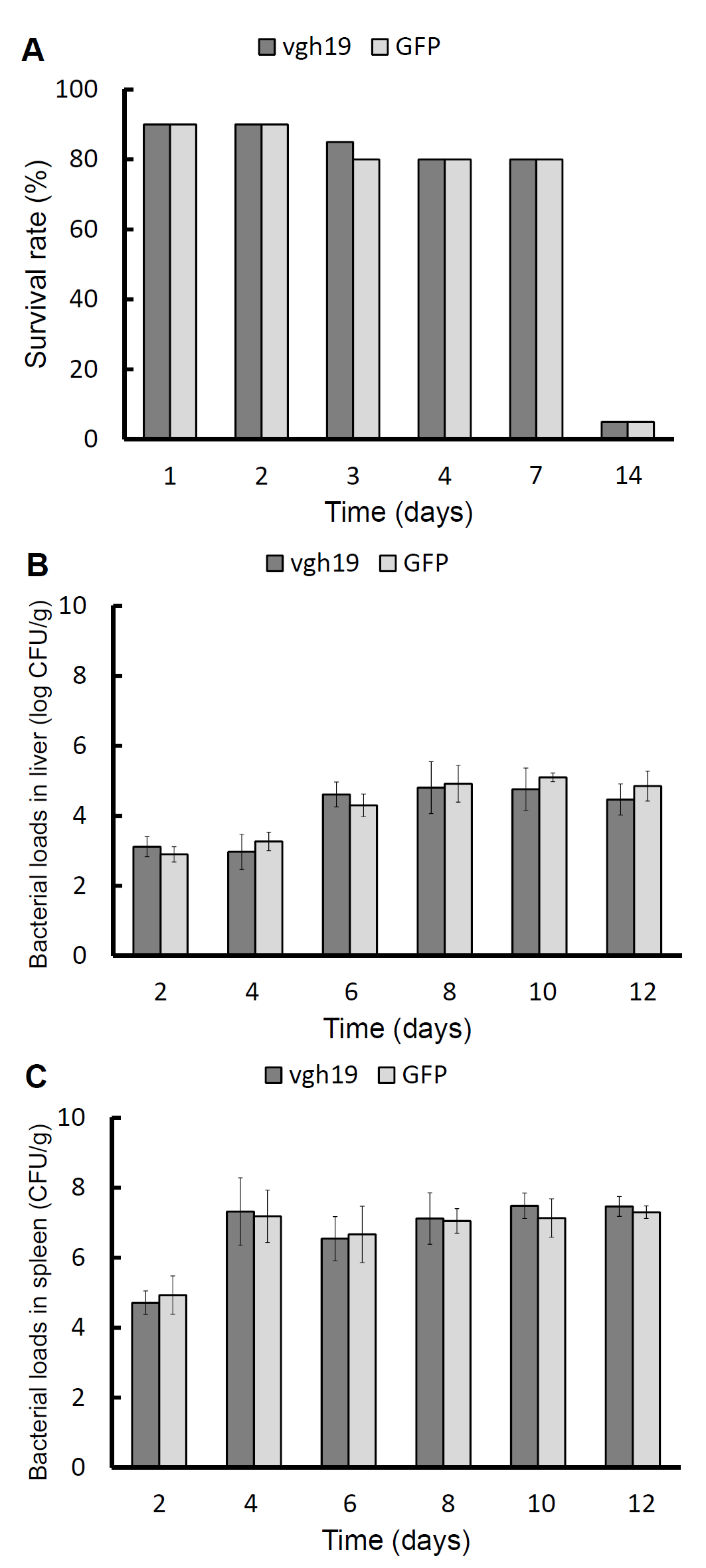

Supplement: Figure S5 — Survival rates (100%) were recorded on the indicated day for BALB/c mice (n = 10, each group) infected with B. pseudomallei vgh19 (50 CFU, dark gray) or GFP (50 CFU, light gray) (A). At the indicated times, individual mice with melioidosis (each experiment, n = 6; duplicate experiments) were sacrificed, and their bacterial burdens in the liver (B) and spleen (C) were determined. The limits of detection were 10 and 250 CFU/g for the liver and spleen, respectively. (TIF) [file pntd.0002363.s005.tif]
