# Supplementary material for: Induction of Mouse Melioidosis with Meningitis by CD11b+ Phagocytic Cells Harboring Intracellular B. pseudomallei as a Trojan Horse
Source: PLoS Negl Trop Dis. 2013 Aug 8;7(8):e2363. doi: 10.1371/journal.pntd.0002363 (PMC3738478; doi:10.1371/journal.pntd.0002363)
Supplement: Table S1 — (DOC) [file pntd.0002363.s006.doc]

| Table S1. Clinical score parameters, assessed values and weight score | | |
| --- | --- | --- |
| Criteria | |  |
| Signs | Assessed value | Weighted score |
| Weight loss from baseline | 5% | 0 |
|  | 10% | 1 |
|  | 15% | 2 |
|  | 20% | 3 |
|  | 25% | 4 |
| Activity | normal | 0 |
|  | increased/decreased | 1 |
|  | mildly diminished | 1 |
|  | diminished | 2 |
|  | severely diminished | 3 |
|  | coma | 4 |
| Time to return to upright position | normal | 0 |
|  | upright < 5 sec | 2 |
|  | upright < 30 sec | 4 |
|  | no return upright | 6 |
| Coat | normal | 0 |
|  | diminished grooming | 1 |
|  | soiled | 1 |
|  | piloerection | 1 |
| Posture | normal | 0 |
|  | slight hunched back | 1 |
|  | severity hunched back | 2 |
| Eyes | normal | 0 |
|  | protruding | 1 |
|  | sunken eyes | 1 |
|  | closed eyelids | 1 |
|  | discharge | 1 |
| Respiration rate (per min) | >150 | 0 |
|  | <150 | 1 |
|  | <100 | 2 |
|  | <75 | 3 |
|  | <50 | 4 |
| Breathing | normal | 0 |
|  | irregular | 2 |
|  | labored | 2 |
| Neurologic exam | normal | 0 |
|  | ataxia | 2 |
|  | limb paresis/ paralysis | 2 |
|  | epileptic seizure | 2 |
|  | status epileptics | 6 |
