# Supplementary material for: Induction of Mouse Melioidosis with Meningitis by CD11b+ Phagocytic Cells Harboring Intracellular B. pseudomallei as a Trojan Horse
Source: PLoS Negl Trop Dis. 2013 Aug 8;7(8):e2363. doi: 10.1371/journal.pntd.0002363 (PMC3738478; doi:10.1371/journal.pntd.0002363)
Supplement: Table S2 — (DOC) [file pntd.0002363.s007.doc]

Table S2. Primary antibodies used for flow cytometry in this study

| Target | Conjugated | Isotype control | Clone | Species | Concentration | Company |
| --- | --- | --- | --- | --- | --- | --- |
| F4/80, | PE | IgG2a κ | BM8 | Rat | 2 μg/ml | Biolegend |
| Ly6C, | PE | IgM κ | AL-21 | Rat | 2 μg/ml | BD Pharmingen |
| Ly-6G, | PE | IgG2a κ | 1A8 | Rat | 2 μg/ml | BD Pharmingen |
| CD3e, | PE | IgG1 κ | 145-2C11 | Hamster | 20 μg/ml | BD Pharmingen |
| CD4 | PE | IgG2a κ | H129.19 | Rat | 2 μg/ml | BD Pharmingen |
| CD8a | PE | IgG2a κ | 53-6.7 | Rat | 2 μg/ml | BD Pharmingen |
| CD19 | PE | IgG2a κ | 1D3 | Rat | 2 μg/ml | BD Pharmingen |
| NK-1.1, | PE | IgG2a κ | PK136 | Mouse | 2 μg/ml | BD Pharmingen |
| CD18, | PE | IgG2a κ | C71/16 | Rat | 2 μg/ml | BD Pharmingen |
| CD31, | PE | IgG2a κ | MEC13.3 | Rat | 2 μg/ml | BD Pharmingen |
| CD11b, | PE-Cy7 | IgG2b κ | M1/70 | Rat | 2 μg/ml | BD Pharmingen |
