# Supplementary material for: Induction of Mouse Melioidosis with Meningitis by CD11b+ Phagocytic Cells Harboring Intracellular B. pseudomallei as a Trojan Horse
Source: PLoS Negl Trop Dis. 2013 Aug 8;7(8):e2363. doi: 10.1371/journal.pntd.0002363 (PMC3738478; doi:10.1371/journal.pntd.0002363)
Supplement: Table S3 — (DOC) [file pntd.0002363.s008.doc]

Table S3. Summary of the characteristics of melioidosis progression during intravenous infection

|  | Melioidosis progress | | |
| --- | --- | --- | --- |
|  | Phase I | Phase II | Phase III |
| Period | day 1 to day 4 | day 5 to day 9 | day 10 to day 15 |
| Mortality^a^ | 20% | 7% | 100% |
| Clinical scores^b^ | 6.56 (5.11-9.6) | 4.2 (3.32-6.1) | 18.5 (17.76-20.47) |
| Signs |  |  |  |
| body weight^c^ | 5-10% | 5-10% | >20% |
| activity | diminished | mildly diminished | severely diminished |
| coat | diminished grooming | diminished grooming | piloerection |
| posture | slight hunched back | slight hunched back | severely hunched back |
| neurological signs | absent | absent | present |
| Cultures^d^ |  |  |  |
| Blood samples | - | -/+ | + |
| CSF samples | - | NA | + |
| Serum cytokines^e^ |  |  |  |
| IFN-γ | ++ | + | ++ |
| IL-6 | ++ | + | ++ |
| IL-10 | + | + | + |
| IL-12 | N | N | N |
| TNF-α | + | + | + |
| MCP-1 | ++ | + | ++ |
| Liver function^f^ |  |  |  |
| GOT | ++ | + | + |
| GPT | ++ | + | + |
| Histology^g^ |  |  |  |
| Abscess occurred in | spleen, liver | spleen, liver, BM | spleen, liver, BM, brain |

a, The number of survivors at the beginning of the indicated phase is considered to be 100% (refer to Figure S3). The injective dose was 50 CFU.

b, Clinical scores on days 4, 6 and 10 post-infection are presented as the mean (95% confidence intervals). The criteria refer to Table S1.

c, loss from baselines

d, Refer to Figure 2. The (+) and (-) symbols represent whether the cultures were positive or negative. NA = not analyzed.

e, Refer to Figure S4A. The (++), (+) and (N) symbols represent highly increased, slightly increased and not increased, respectively.

f, Refer to Figure S4B. The ++, + and N symbols represent highly increased, slightly increased and not increased.

g, Refer to Figure 1.
