# Supplementary material for: Induction of Mouse Melioidosis with Meningitis by CD11b+ Phagocytic Cells Harboring Intracellular B. pseudomallei as a Trojan Horse
Source: PLoS Negl Trop Dis. 2013 Aug 8;7(8):e2363. doi: 10.1371/journal.pntd.0002363 (PMC3738478; doi:10.1371/journal.pntd.0002363)
Supplement: Table S4 — (DOC) [file pntd.0002363.s009.doc]

Table S4. Summary of the characteristics of melioidosis progression after adoptive transfer

|  | Melioidosis progress after adoptively transferred by^a^ | | | | Control sera^f^ |
| --- | --- | --- | --- | --- | --- |
|  | CD11b^+^ | CD11b^-^ | Selectin-expressing | Selectin-negative |  |
| Clinical scores^b^ | 11.3 (10.6-14.3) | 4.5 (4.2-5.8) | 12.5 (11.6-15.9) | 2.5 (1.7-5.7) |  |
| Histological exam. |  |  |  |  |  |
| Meninges^c^ | I or M | R or N | I or M | R or N |  |
| Abscess in | Spleen, Liver, BM | Spleen, Liver, BM | Spleen, Liver, BM | Spleen, Liver, BM |  |
| Bacteriological exam.^d^ |  |  |  |  |  |
| CSF cultures | Positive | Negative | Positive | Negative |  |
| Bacterial loads in |  |  |  |  |  |
| Brain (CFU/g) | 3.4±0.6 x 10^3^ | < 5 | 2.0±1.5 x 10^3^ | < 5 |  |
| Liver (CFU/g) | 1.8±0.7 x 10^6^ | 5.9±2.1 x 10^5^ | 1.1±0.6 x 10^6^ | 5.5±1.8 x 10^5^ |  |
| Spleen (CFU/g) | 1.5±0.6 x 10^6^ | 2.1±0.7 x 10^6^ | 2.0±1.4 x 10^6^ | 1.1±0.2 x 10^6^ |  |
| BM (CFU/10^6^ cells) | 4.1±4.0 x 10^3^ | 3.0±2.0 x 10^3^ | 1.6±1.1 x 10^4^ | 4.3±3.7 x 10^3^ |  |
| Serum cytokines (pg/ml) |  |  |  |  |  |
| TNF-α | 835±209 | NA^e^ | 174±123 | 29±20 | 18±4 |
| IL-1β | 118±60 | NA | 48±25 | 13±4 | 8±1 |
| IL-6 | 9525±119 | NA | 3862±2872 | 370±246 | 26±12 |
| IFN-γ | 4996±364 | NA | 326±95 | 635±119 | 28±7 |
| MCP-1 | 5147±768 | NA | 6273±1353 | 3242±545 | 37±12 |
| Liver function (IU/ml) |  |  |  |  |  |
| GOT | 1472±159 | NA | 1827±815 | 670±145 | 70±9 |
| GPT | 1153±130 | NA | 1061±697 | 280±62 | 10±2 |

a, The donor cells were adjusted to contain 2000 CFU intracellular *B. pseudomallei* (see materials and methods). The data in this table were collected from the mice on day 2 post-transfer.

b, The clinical scores are represented as the mean (95% confidence intervals). The criteria refer to Supplemental Table 1.

c, By histological examination, intensive (I), moderate (M), rare (R) or no (N) neutrophil infiltration occurred in meninges (Refer to Figure 8B).

d, Refer to Figure 8D.

e, Not analyzed.

f. Control sera obtained from healthy mice as the baseline.
