# Supplementary material for: Induction of Mouse Melioidosis with Meningitis by CD11b+ Phagocytic Cells Harboring Intracellular B. pseudomallei as a Trojan Horse
Source: PLoS Negl Trop Dis. 2013 Aug 8;7(8):e2363. doi: 10.1371/journal.pntd.0002363 (PMC3738478; doi:10.1371/journal.pntd.0002363)
Supplement: Text S1 — (DOC) [file pntd.0002363.s010.doc]

Text S1. Protocols and comments of adjusting donor cells

The protocols of adjustment of donor cells included three parts: preliminary test, determination of the ratio of bacteria to GFP and adjustment (refer to Figure S2).

A. Preliminary test

The donor cells were respectively performed three experiments: microscopic observation, flow cytometric analysis and bacterial counts on plate after serial dilution.

Step 1, Under microscopic observation, the intracellular GFP cells were divided into three bins: low (*ca.* 1 rod), intermediate (*ca.* 5 rods) and high (*ca.* ≥10 rods) levels [Comment A].

Step 2, The distribution of the isolated cells (n=300) at low (L%), intermediate (I%) and high (H%) levels was calculated [Comment B].

Step 3, By flow cytometric analysis, the number of fluorescent (F) cells was determined in the isolated population (5x10^4^ cells).

Step 4, Based on the distribution of each level (as determined above *via* microscopic observation), the fluorescent cells were reassigned into groups based on their level (low, intermediate and high) [Comment C].

Step 5, Total amounts of intracellular bacteria (GFP cells) were determined in the fluorescent cells.

Step 6, Viable intracellular bacteria were enumerated using bacterial plate counts after serial dilution [Comment D]. The ratios of viable *B. pseudomallei* to fluorescent cells and to GFP cells were calculated

B. Determination of the ratio of bacteria to GFP

Step 7, The ratio of viable *B. pseudomallei* to intracellular GFP cells in each donor cell was measured. Approximately 3-8 CFU of *B. pseudomallei* were cultivated from the donor cells that contained 1000 GFP cells [Comment E].

C. Adjustment

Step 8, By flow cytometric analysis, the numbers of fluorescent cells were steadily determined from a new preparation of adoptive transfer.

Step 9, The donor cells were adjusted to carry 50, 500 or 2000 CFU in accordance with the above preliminary data (i.e., the ratio of viable *B. pseudomallei* to fluorescent cells) [Comment F].

Step 10, The data of adoptive transfer experiments were discarded if intracellular *B. pseudomallei* within the donor cells were determined to be >± 15% of 50, 500 or 2000 CFU based on bacterial counts. [Comment G].

**Comments:**

A. The amount of GFP within cells reflected the number of intracellular *B. pseudomallei*.

B. A fluorescence microscope (Eclipse 50i; Nikon, Shinjuku, Tokyo, Japan) and imaging software (NIS-Elements D3.2; Nikon) were used.

C. One fluorescent cell most likely contained more than one GFP cell. In general, the distribution of each level differed depending on the population. For example, 51.3% low, 22% intermediate and 26.7% high level cells were observed for the BM CD11b^+^ population, whereas 82% low, 18% intermediate and 0% high level cells were observed in the BM CD11b^-^ population.

D. Many confounding factors such as intrinsic autofluorescence, cell plasticity and the complexities of granulocytic compartmentalization as well as inactive or uncultivable bacteria carrying GFP, a lack of infectivity would result in false positive results. Thus, the amounts of viable *B. pseudomallei* were determined by bacterial plate counts after serial dilution.

E. The results showing that the ratio of viable *B. pseudomallei* to GFP cells was reproducible suggest that the fluorescent cells from every preparation reflect the amounts of viable intracellular *B. pseudomallei*.

F. The concentration of donor cells ranged from 10^4^ to 10^6^ cells. For example, the concentrations of BM CD11b^+^ donor cells were as follows: 1x10^4^ cells for 50 CFU, 1x10^5^ cells for 500 CFU and 4x10^5^ for 2000 CFU. The concentrations of BM CD11b^-^ donor cells were as follows: 2.5x10^4^ for 50 CFU, 2.5x10^5^ for 500 CFU and 1x10^6^ for 2000 CFU.

G. The estimation of intracellular *B. pseudomallei* using the ratio of viable *B. pseudomallei* to fluorescent cells corresponded with the bacterial plate counts in >90% of the preparations.
